# The chronic wound milieu changes essential oils' antibiofilm activity—an in vitro and larval model study

**DOI:** 10.1038/s41598-024-52424-6

**Published:** 2024-01-26

**Authors:** Malwina Brożyna, Bartłomiej Dudek, Weronika Kozłowska, Katarzyna Malec, Justyna Paleczny, Jerzy Detyna, Krystyna Fabianowska-Majewska, Adam Junka

**Affiliations:** 1 Platform for Unique Models Application, Department of Pharmaceutical Microbiology and Parasitology, Wroclaw Medical University, Wroclaw, Poland; 2 Division of Pharmaceutical Biotechnology, Department of Pharmaceutical Biology and Biotechnology, Wroclaw Medical University, Wroclaw, Poland; 3 Department of Drug Form Technology, Wroclaw Medical University, Wroclaw, Poland; 4 Department of Mechanics, Materials and Biomedical Engineering, Wroclaw University of Science and Technology, Wroclaw, Poland; 5 Faculty of Medicine, Lazarski University, Warsaw, Poland

**Keywords:** Antimicrobials, Bacteria, Biofilms, Pathogens

## Abstract

Essential Oils (EOs) are currently being researched as potential antibiofilm agents to combat infections related to chronic wound biofilms. As documented in the literature, EOs’ in vitro antibacterial properties are often assessed using standard microbiological media and conditions that do not accurately reflect the actual environment of a chronic wound. To address this issue, In vitro Wound Milieu (IVWM) medium, which closely resembles the environment of a chronic wound, was applied for culturing *S. aureus* biofilms (n = 12) in this research. Biofilms cultivated in the standard Tryptic Soy Broth (TSB) medium served as a control for the experiment. Key biofilm features were analyzed and compared. Subsequently, staphylococci were exposed to the activity of thyme or rosemary EOs (T-EO and R-EO, respectively). As proof of concept, the cytotoxicity of T-EO and its antimicrobial in vivo activity were assessed using a *G. mellonella* larvae model. Key features of biofilm-forming cells were lower in the IVWM than in the TSB medium: biomass (up to 8 times), metabolic activity (up to 9 times), cell number (up to 100 times), and the live/dead cells ratio. Conversely, biofilm thickness was higher (up to 25%) in IVWM. These differences translated into varied responses of the biofilms to EOs exposure. The application of T-EO led to a greater reduction (up to 2 times) in 67% of biofilm-forming strains in IVWM compared to the TSB medium. Conversely, exposure to R-EO resulted in a higher reduction (up to 2.6 times) of 83% of biofilm-forming strains in TSB than in IVWM. The application of T-EO was not only non-toxic to *G. mellonella* larvae but also increased the survival of larvae infected with staphylococci (from 48 to 85%). Our findings suggest that EOs not only show promise as agents for treating biofilm-related wound infections but also that providing conditions reflecting the specific niche of the human body is of paramount importance in influencing the results obtained. However, before clinical application, challenges related to the methods of assessing their activity, microbial intra-species variability, and different levels of activity of various EOs should be analyzed and standardized.

## Introduction

The dissemination of antibiotic-resistant microorganisms poses a global threat to public health care. As the efficacy of existing antibiotics decreases and new ones remain undeveloped, the applicability of non-antibiotic therapeutics to combat infections is being thoroughly investigated worldwide^1^.

Non-healing wounds are particularly prone to infections caused by antibiotic-resistant microorganisms^2^. These wounds affect 20 million patients annually and require more than 31 billion USD per year for treatment^3^. The five-year mortality rate for people with diabetic non-healing wounds is comparable to the five-year mortality rate for patients with cancer (30.5% vs. 31%, respectively)^4^.

Infections, one of the most frequent complications of non-healing wounds, are caused by biofilms – complex microbial communities embedded within an extracellular matrix (ECM)^5^. The ECM provides not only a physical barrier to antimicrobial agents but also protects against the immune response. Furthermore, some microbial cells within the biofilm matrix exhibit low cellular activity, rendering them insensitive to antibiotics that target cellular transcription or translation processes^6^. In general, biofilms exhibit significantly higher tolerance to the immune system and antimicrobials compared to their free-floating (planktonic) counterparts.

Thus, the treatment of infected, non-healing wounds is a major challenge for modern medicine. Current strategies to eradicate wound biofilms involve the debridement (removal) of infected tissue, combined with the topical application of dressings and antiseptic agents^7^. Topical administration of antibiotics is no longer recommended due to their low activity against biofilms and the potential to induce microbial resistance, hypersensitivity, or contact allergy^8^.

Modern antiseptic agents are considered first-line antimicrobials for non-healing wound management due to their broad spectrum of antimicrobial activity, non-specific mode of action, and low in vivo cytotoxicity^9^. However, several reports indicate the potential for microorganisms to develop tolerance to modern antiseptic agents (compounds obtained through industrial chemical synthesis). The survival of *Burkholderia cepacia* in octenidine dihydrochloride-containing solutions and the increased tolerance of certain strains of *Pseudomonas aeruginosa* to octenidine dihydrochloride exemplify such risks^10,11^.

It can be inferred that the effectiveness of systemic antibiotic therapy against wound biofilms is limited, and the topical administration of these antimicrobials is not recommended. The use of modern antiseptic agents (e.g., polyhexanide, povidone-iodine) still correlates with favorable clinical outcomes, but the example of octenidine dihydrochloride raises concerns that the efficacy of these antiseptics may also be reduced due to microorganisms' tendency to develop resistance^10,11^. Therefore, not only non-antibiotic but also non-antiseptic approaches are now considered next-generation strategies for treating biofilm-related infections. These methods include the use of natural compounds, enzymes, and other bioactive molecules that can disrupt biofilm formation and/or enhance host immune responses^12^. In this regard, plant-derived Essential Oils (EOs) are of great interest as potential antibiofilm agents. These multi-component volatile liquids exhibit a broad spectrum of antimicrobial activity against Gram-positive and Gram-negative bacteria and fungi^13^. EOs can also interfere with or impede various processes occurring in biofilms, such as adhesion, quorum-sensing, or modulation of the expression of biofilm-related genes^14^.

The non-specific mode of EOs' antimicrobial action is considered to limit the risk of triggering bacterial resistance^15^. Thanks to their low cytotoxicity, anti-inflammatory activity, and ability to promote the wound healing process, the use of EOs can be perceived as an effective future strategy for the treatment of non-healing wound infections^16^. Although several in vitro studies report significant antimicrobial properties of EOs, these likely do not reflect their actual in vivo activity^17–19^. Major shortcomings of this in vitro research include using standard microbiological media (rather than media reflecting the composition of wound exudate) and the evaluation of EOs' activity primarily against planktonic forms of microbes, not against biofilms. Recent reports indicate that providing a milieu that mimics the wound environment in in vitro tests significantly alters key biofilm characteristics such as metabolic activity, three-dimensional structure, and matrix composition, thereby affecting its tolerance to antimicrobial agents^20,21^. Therefore, in this study, we cultivated a *Staphylococcus aureus* biofilm (one of the main factors of wound infection) in a medium formulated in 2021 by Kadam et al., known as the In vitro Wound Milieu (IVWM), which comprises serum, cell–matrix elements, and host factors that reflect the wound environment^22^. After conducting a thorough analysis of the key properties of the biofilm grown in IVWM and comparing them to those of the biofilm cultivated in standard microbiological Tryptic Soy Broth (TSB) medium, we exposed staphylococcal biofilms to the activity of thyme and rosemary EOs (T-EO, R-EO, respectively). We also carried out control analyses on planktonic *Staphylococcus aureus* cells. Moreover, the model with *Galleria mellonella* larvae was performed to assess T-EO cytotoxicity and confirm this Essential Oil’s anti-staphylococcal activity in vivo. To the best of our knowledge, this is the first study that assesses the antimicrobial properties of EOs under conditions that resemble a chronic wound milieu.

## Results

### Assessment of EOs chemical composition

In the first line of the experiment, a GC–MS analysis was performed to evaluate the percentage composition of the EOs’ components. T-EO comprised 50.6% thymol, 19.2% p-cymene, and 9.1% γ-terpinene. Three main components of R-EO were: 21.1% α-pinene, 20.0% 1,8-cineole, and 18.5% camphor. A detailed list of the composition of the EOs is presented in Supplementary Table 1.

### Evaluation of biofilm biomass, biofilm metabolic activity, and the number of colony-forming units

**Figure 1:**
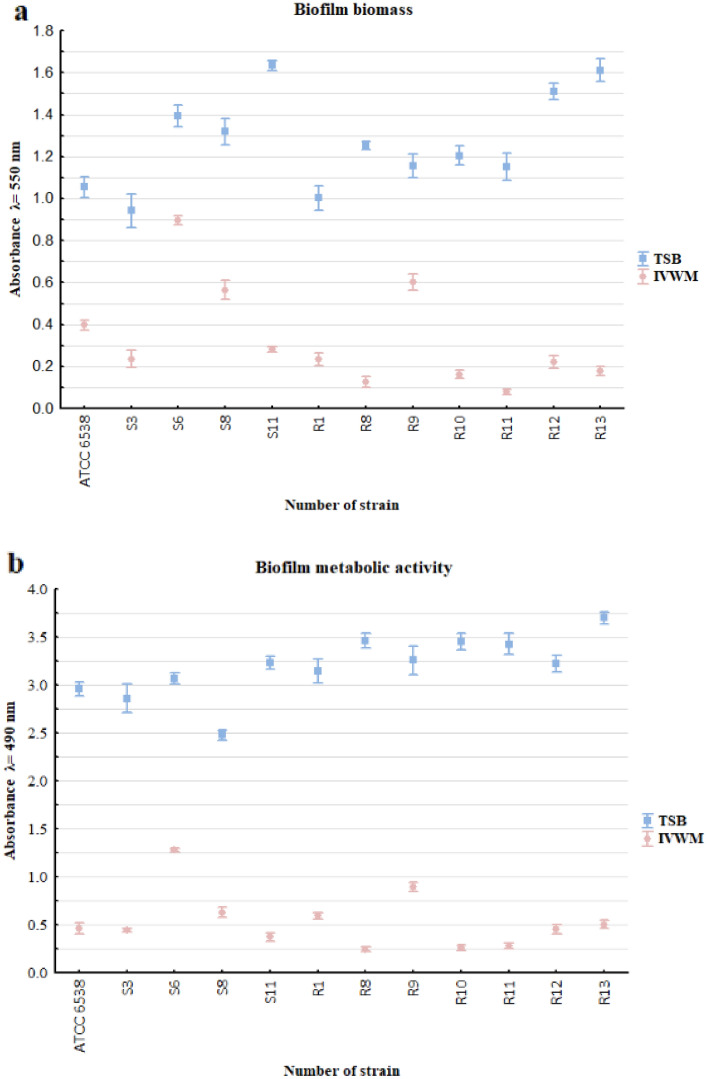
The average biofilm biomass (**a**) and metabolic activity (**b**) of analyzed *S. aureus* strains (n = 12) cultured in TSB or IVWM medium. Differences between the two media were statistically significant for all strains (*p* < 0.05, *t*-test or Welch’s *t*-test for biofilm biomass assay, *t*-test or Welch’s *t*-test or Mann–Whitney U test for the biofilm metabolic activity assay). The error lines represent the standard error of the mean.

The ability of *S. aureus* strains to form biofilm biomass in standard TSB or IVWM medium was evaluated using the crystal violet method (CV). The biofilms’ metabolic activity was assessed using tetrazolium chloride (TTC) staining (Fig. 1).

**Figure 2:**
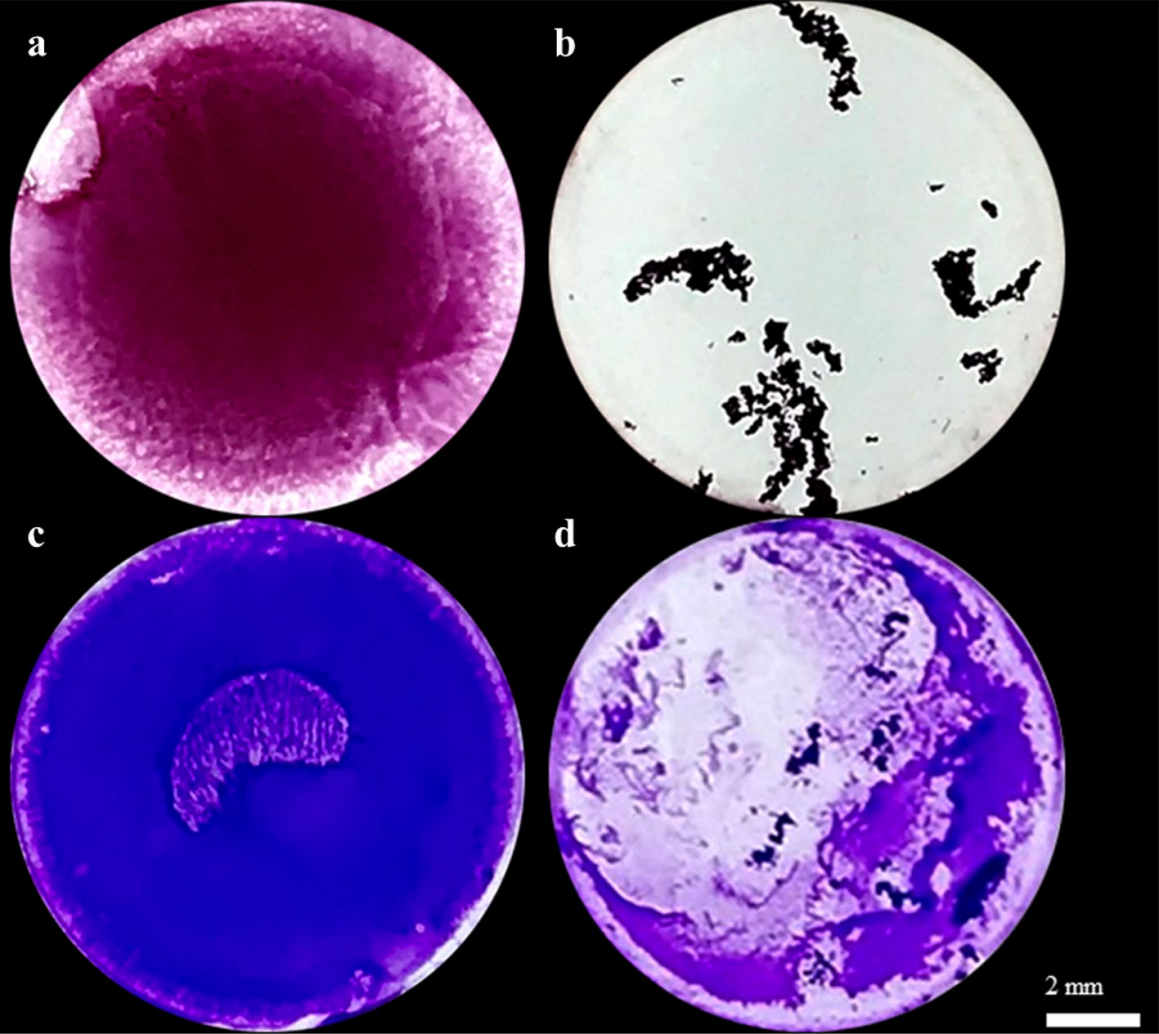
Macroscopic visualization of representative changes in the features of biofilm cultivated in TSB or IVWM medium, exemplified by *S. aureus* S11 biofilm formed on polystyrene. Panels a and b depict biofilm cultured in TSB or IVWM, respectively, and stained with tetrazolium chloride; Panels c and d show biofilm cultured in TSB or IVWM, respectively, and stained with crystal violet. The scale bar represents 2 mm.

All strains were able to form a biofilm in TSB or IVWM medium. The biomass and metabolic activity of all biofilms were higher in TSB than in IVWM medium. For all strains, this trend was statistically significant (*p* < 0.05, *t*-test or Welch’s *t*-test for the biofilm biomass assay, *t*-test or Welch’s *t*-test or Mann–Whitney U test for the biofilm metabolic activity assay, Supplementary Tables 2, 3 and Supplementary Figures 1, 2), and it also remained statistically significant when the mean of all strains was calculated (*p* = 0.000000, Mann–Whitney U test), Supplementary Figure 3, Supplementary Table 4). A significant linear correlation (*p* = 0.001) between the level of biofilm biomass and metabolic activity for biofilms cultured in IVWM medium, but not in TSB medium (*p* = 0.315), was observed (Supplementary Table 5, Supplementary Figure 4). The correlation coefficient was determined as moderate or very strong for TSB (r = 0.32) or IVWM (r = 0.83), respectively. To confirm data obtained by semiquantitative CV and TTC methods, the number of biofilm-forming cells of reference strain cultivated in TSB or IVWM was quantitatively cultured (Table 1). The average number of biofilm-forming cells was significantly higher (*p* = 0.001124, *t*-test, Supplementary Table 6, Supplementary Figure 5A,B) in TSB than IVWM medium. A macroscopic visualization of representative biofilm stained with crystal violet or tetrazolium chloride confirming differences in the level of biofilm biomass and metabolic activity between bacteria cultured in TSB or IVWM is presented in Fig. 2. The biofilm in the TSB was highly confluent, i.e., biofilm-forming cells covered essentially the entire surface of the plate’s wells (Fig. 2a,c). Biofilm cells cultured in IVWM formed cells aggregates unequally distributed on the well’s surface (Fig. 2b,d).

**Table 1:** A mean number of Colony-Forming Unit (CFU/mL) of *S. aureus* ATCC 6538 biofilm cultured in TSB or the IVWM medium. SEM- standard error of the mean. Differences between the two media were statistically significant (*p* = 0.001124, *t*-test).

Number of CFU/mL				
Strain	TSB		IVWM	
ATCC 6538	Mean	SEM	Mean	SEM
	8.98E + 14	1.07E + 14	1.46E + 12	6.65E + 11

### Visualization of biofilm-forming cells

**Figure 3:**
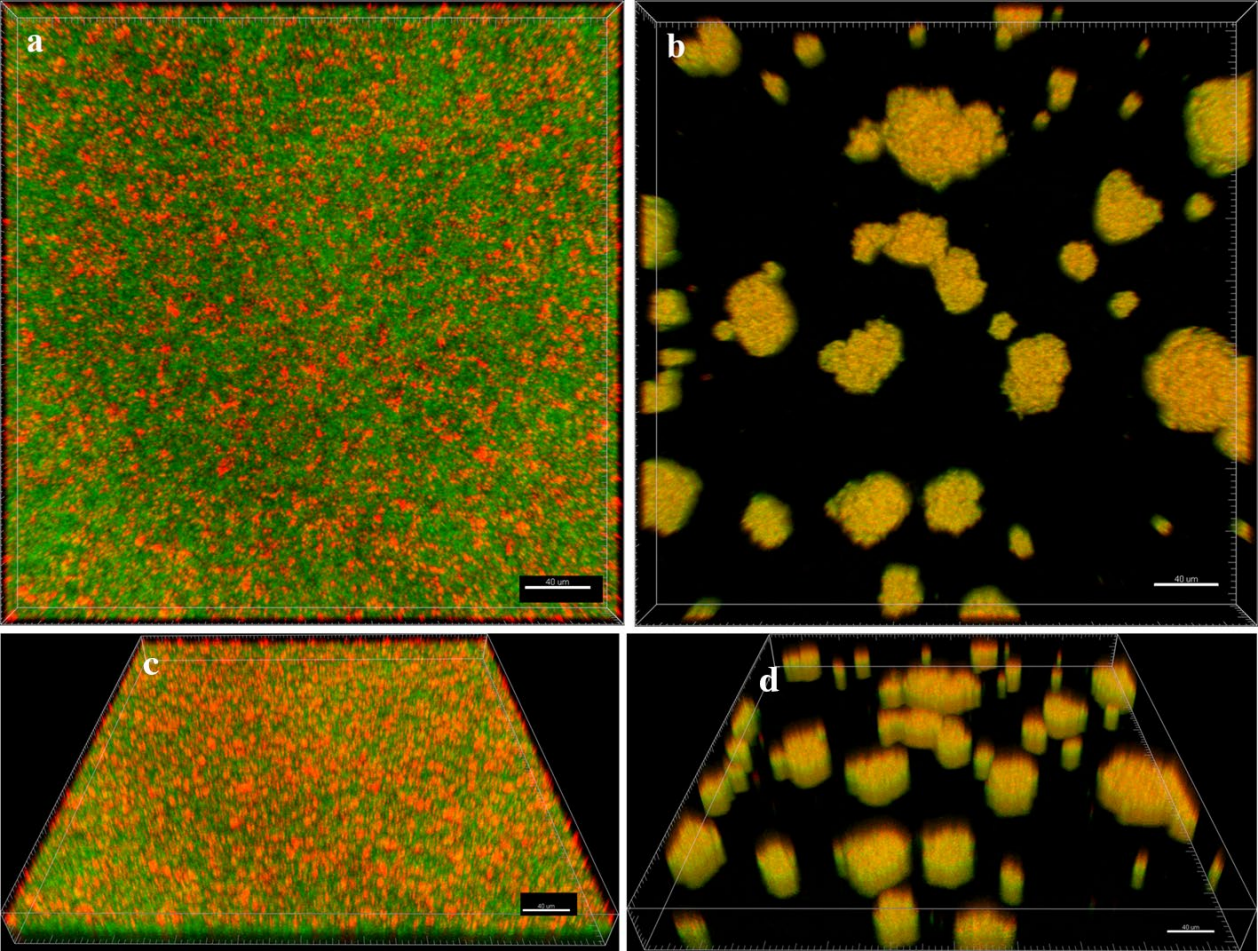
Microscopic visualizations of the *S. aureus* biofilm formed on polystyrene and stained with a LIVE/DEAD dye. (**a**, **b**) an aerial perspective of the S11 biofilm cultured in TSB (**a**) or IVWM (**b**); (**c**, **d**) the Z- axis image stack visualizing the S11 biofilm cultured in TSB (**c**) or IVWM (**d**) from the side aerial perspective. The red/orange color indicates staphylococcal cells of altered/damaged cell walls, while green-colored shapes show unaltered cell walls. The scale bar is 40 µm. The confocal microscope SP8, magnification 25 ×.

**Figure 4:**
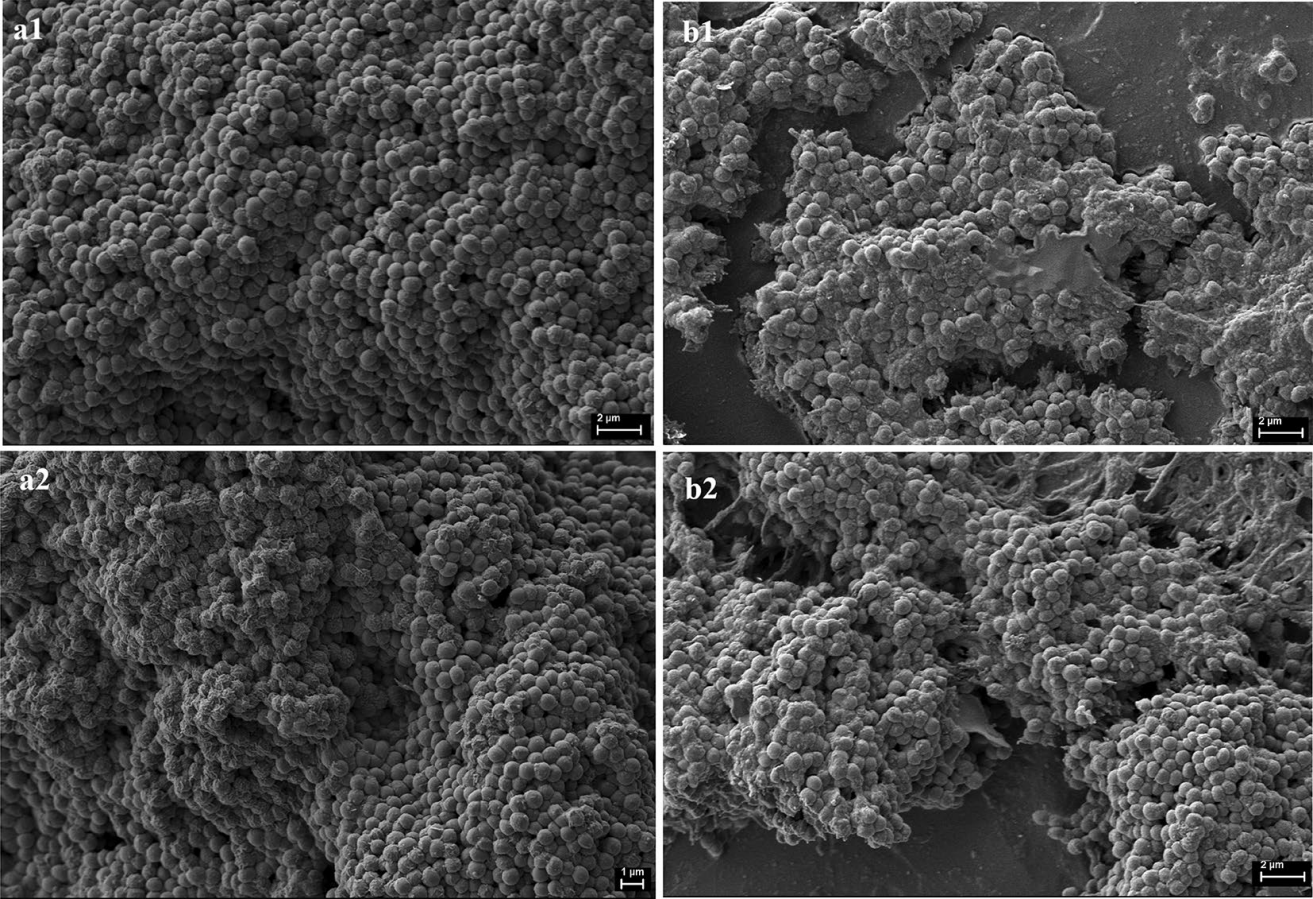
Microscopic visualizations of the *S. aureus* ATCC 6538 (**a1**, **b1**) or R1 (**a2**, **b2**) biofilm formed on agar and cultured in TSB (**a1**, **a2**) or IVWM (**b1**, **b2**) medium, respectively. The scale bar is 1 or 2 µm. Scanning Electron Microscope Zeiss Auriga 60 (magnification 10.00 KX).

Next, the analysis of live and dead cells within the Z-axis (thickness) of the 24-h-old staphylococcal biofilm structure was performed using LIVE/DEAD (L/D) dyeing and confocal microscopy (Fig. 3). The staphylococcal biofilm cultured in TSB resembled the structure of a dense lawn (Fig. 3a,c). The biofilm-forming cells evenly covered the whole field of vision. In turn, the biofilm cultivated in IVWM formed bubble-like structures of diversified sizes (Fig. 3b,d). The IVWM biofilm contained regions of differential thickness and cell density, including areas not covered with cells (black regions). Structural differences between biofilms cultured in TSB or IVWM medium were also observed in microscopic visualizations performed with the Scanning Electron Microscope (SEM) (Fig. 4).

**Figure 5:**
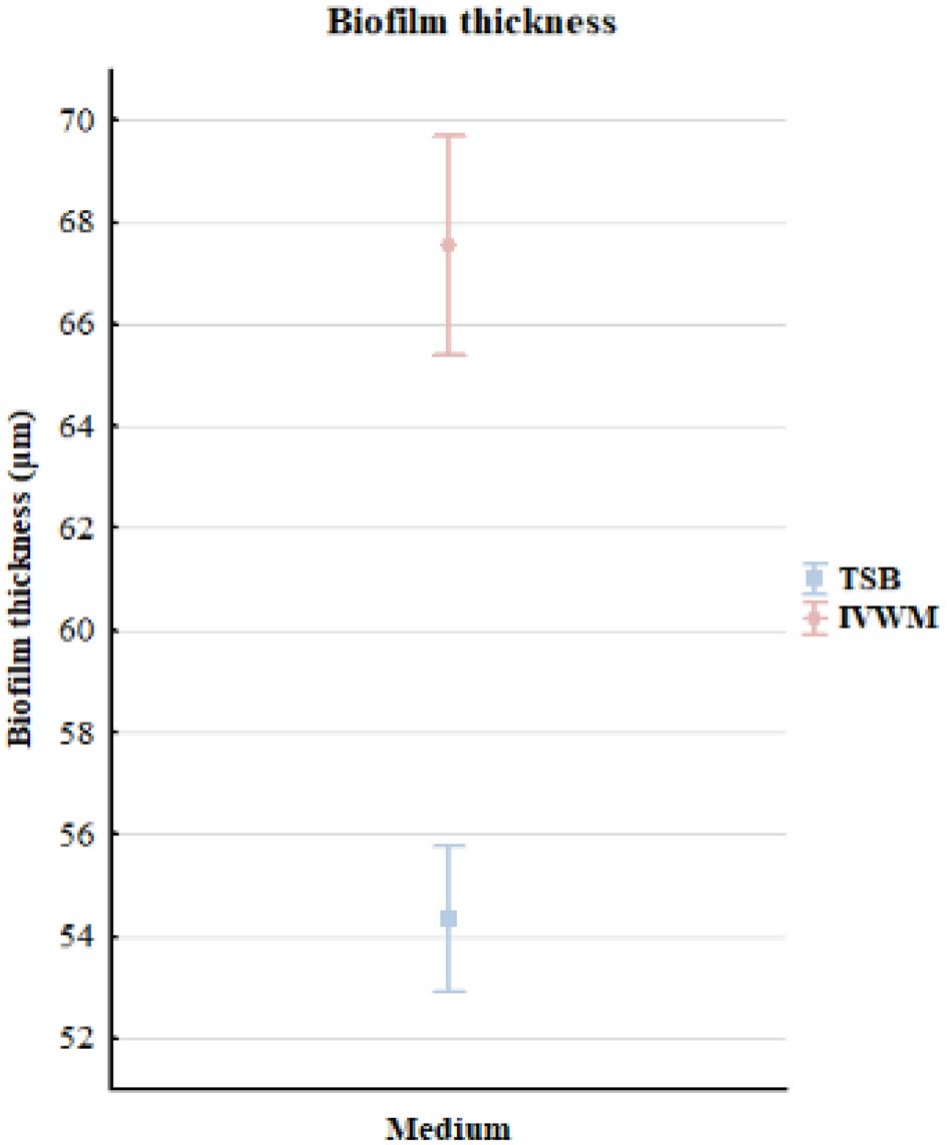
Comparison of the average thickness (µm) of all staphylococcal biofilms (n = 12) cultured in TSB or IVWM medium measured with the confocal microscopy. The error lines represent the standard error of the mean (*p* = 0.000006, Mann–Whitney U test).

The average thickness of the biofilm formed by all *S. aureus* strains in TSB medium was significantly lower than in IVWM medium (*p* = 0.000006, Mann–Whitney U test, Fig. 5, Supplementary Table 6, Supplementary Figure 5C,D).

In turn, the share of live cells in biofilm was higher for ten (83% of strains) strains cultured in TSB medium than in IVWM (Table 2). The average share of live cells was also significantly higher (*p* = 0.000003, Mann–Whitney U test) in TSB than in IVWM when all strains were analyzed together (Supplementary Table 6, Supplementary Figure 5E,F,G).

**Table 2:** Average percentage (%) share of live and dead cells in all biofilm-forming staphylococcal strains (n = 12) cultured in TSB or IVWM medium measured with the confocal microscope. SEM- standard error of the mean.

Percentage (%) share of cells in *S. aureus* biofilms						
Strain number	TSB			IVWM		
	LIVE	DEAD	SEM	LIVE	DEAD	SEM
ATCC 6538	84	16	0.1	80	20	1.0
S3	83	17	0.5	73	27	0.9
S6	76	24	2.3	61	39	3.1
S8	79	21	3.3	73	27	1.8
S11	74	26	2.4	63	37	1.5
R1	82	18	0.5	54	46	3.2
R8	86	14	1.7	67	33	0.5
R9	86	14	0.4	76	24	1.4
R10	66	34	1.1	68	32	0.9
R11	71	29	2.2	69	31	0.5
R12	70	30	0.5	74	26	1.3
R13	82	18	0.6	47	53	5.4

**Figure 6:**
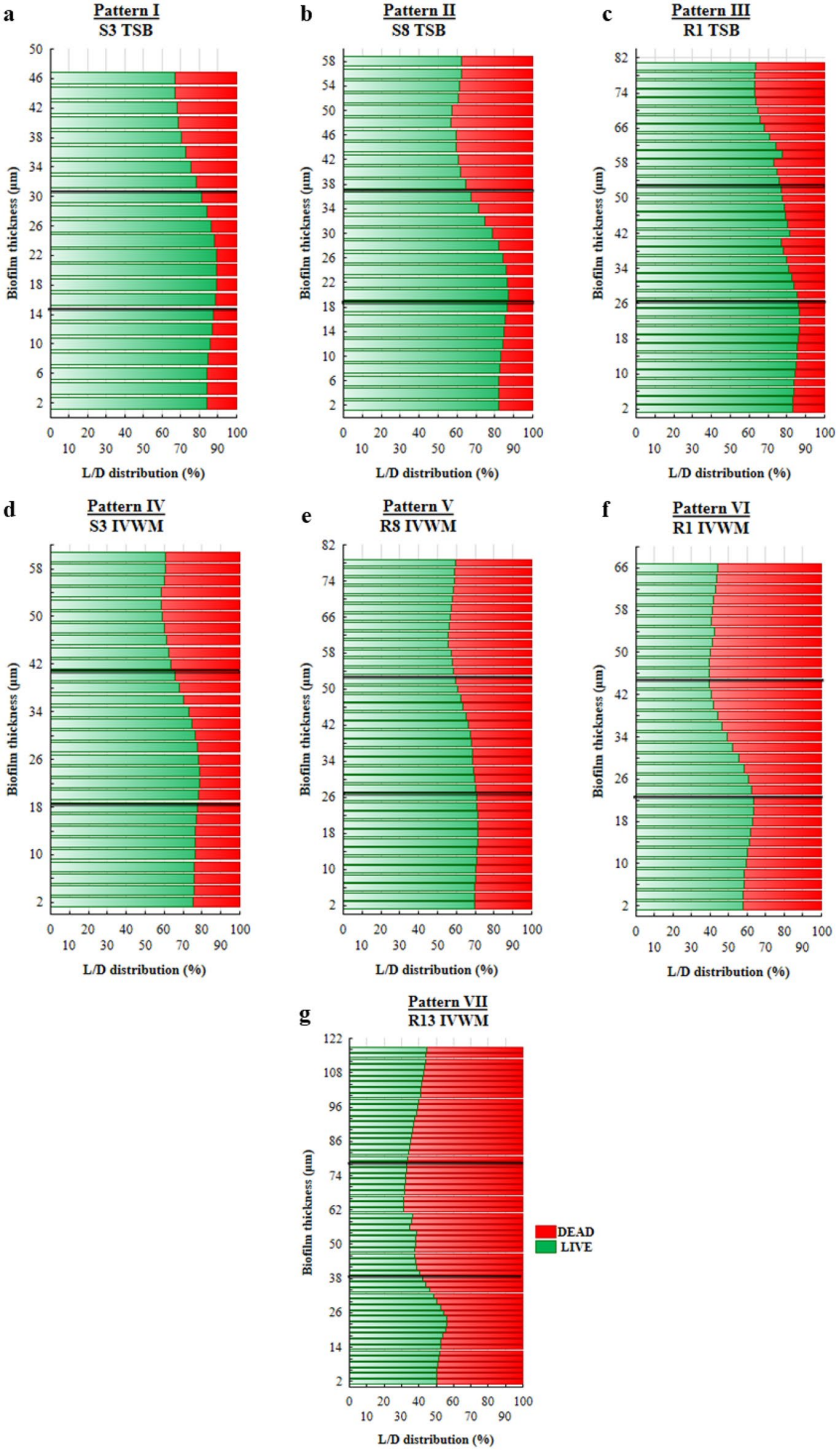
The main patterns of live (L, green) and dead (D, red) cells distribution (%) in staphylococcal biofilms across the Z-axis cultured in TSB or IVWM medium. The thickness of each section was 2 µm. Black lines divide graphs into the top (T), middle (M), and bottom (B) parts. I (**a**), II (**b**), and III (**c**) strains representing particular patterns of cells share in TSB; IV (**d**), V (**e**), VI (**f**), and VII (**g**) strains representing particular patterns of cells share in IVWM. S3, S8, R1, R8, R13- *S. aureus* clinical strains. Figures of the other strains are presented in Supplementary Figure 6. The confocal microscope SP8, magnification 25 × .

The analysis of the cellular spatial composition of the biofilms allowed us to distinguish their three sections: bottom one (B, the closest to the polystyrene surface), middle (M), and top (T, the farthest from the polystyrene surface) (Figs. 3 and 6, Supplementary Figure 6, Supplementary Table 7). Different patterns of live (green) and dead (red) share in parts (T, M, and B) of biofilms were revealed in each medium. Three (I, II, III) patterns were distinguished in TSB medium, and four (IV, V, VI, VII) in IVWM (Fig. 6, Supplementary Figure 6). The share of dead cells was higher in the T parts than in the M and B parts in 100% or 92% of staphylococcal biofilms grown in TSB or IVWM medium, respectively (Fig. 6, Supplementary Figure 6, Supplementary Table 7). A higher share of live cells was observed in B (for 92% of strains) and M (for 83% of strains) parts of biofilms cultured in TSB medium than in IVWM (Fig. 6, Supplementary Figure 6, Supplementary Table 7). For 58% of staphylococcal biofilms, the share of live cells in T parts was higher in TSB medium than in IVWM.

### Analysis of the size of EOs emulsion droplets

The average diameters of the EOs droplets within the prepared formulations were measured. The droplets of T-EO and R-EO were 209 ± 22 nm and 995 ± 341 nm, respectively. The polydispersity index (PDI) of T-EO and R-EO’s emulsions was 0.46 and 0.68, respectively.

### Evaluation of antimicrobial and antibiofilm activity of EOs

The antimicrobial activity of the emulsified T- and R-EOs against planktonic *S. aureus* cells was evaluated using a microdilution technique (Table 3). The applied emulsifier, Tween 20, did not affect the viability of staphylococcal cells (Supplementary Figure 7). The Minimal Inhibitory Concentrations (MIC) values of applied EOs were higher for 75% (T-EO) or 58% (R-EO) of strains cultivated in IVWM compared to strains cultivated in TSB medium. T-EO acted 2 to 4 times weaker against this 75% of strains in IVWM than in TSB medium. For 50% of strains treated with R-EO, the MIC values were twice higher in IVWM than in TSB, and for 8%, this parameter was sixty-four times higher in IVWM than in TSB.

**Table 3:** Antimicrobial activity of the tested EOs’ emulsions against planktonic (MIC (%) (v/v)) and biofilm cells (MBEC (%) (v/v)) of reference or clinical strains of *S. aureus*. N/R indicates EOs where MBEC values were not reached at the highest applied concentration (10% (v/v)) of R-EO. T-EO—thyme Essential Oil, R-EO—rosemary Essential Oil, TSB—Tryptic Soy Broth, IVWM—In vitro Wound Milieu, MIC—Minimal Inhibitory Concentration, MBEC—Minimal Biofilm Eradication Concentration.

	MIC (%)				MBEC (%)			
	T-EO		R-EO		T-EO		R-EO	
Strain number	TSB	IVWM	TSB	IVWM	TSB	IVWM	TSB	IVWM
ATCC 6538	0.02	0.04	1.25	1.25	0.63	0.63	N/R	N/R
S3	0.16	0.31	1.25	2.5	0.63	0.63	2.5	N/R
S6	0.31	0.31	1.25	2.5	0.63	0.63	N/R	N/R
S8	0.16	0.31	2.5	2.5	0.63	0.63	N/R	N/R
S11	0.08	0.16	1.25	1.25	0.63	0.63	N/R	N/R
R1	0.31	0.31	1.25	2.5	1.25	0.63	N/R	N/R
R8	0.16	0.31	0.04	2.5	1.25	2.5	N/R	N/R
R9	0.16	0.31	1.25	1.25	1.25	0.63	N/R	N/R
R10	0.16	0.16	1.25	2.5	0.63	0.63	N/R	N/R
R11	0.16	0.31	1.25	2.5	0.63	1.25	N/R	N/R
R12	0.04	0.16	2.5	2.5	1.25	0.63	N/R	N/R
R13	0.08	0.31	1.25	2.5	1.25	2.5	N/R	N/R

In the case of biofilms, T-EO exhibited higher activity than R-EO regardless of the medium applied (Table 3). The MBEC (Minimal Biofilm Eradication Concentration) values of T-EO were determined for all staphylococcal strains and ranged from 2.5% (v/v) to 0.63% (v/v). The MBEC value of T-EO differed in 50% of strains, depending on the medium applied (TSB or IVWM). For half of these strains, the MBEC value was higher in IVWM than in TSB medium. In the case of R-EO, no MBEC values were achieved; therefore, the assessment of the biofilm cells reduction (%) was performed in the subsequent analyses.

**Figure 7:**
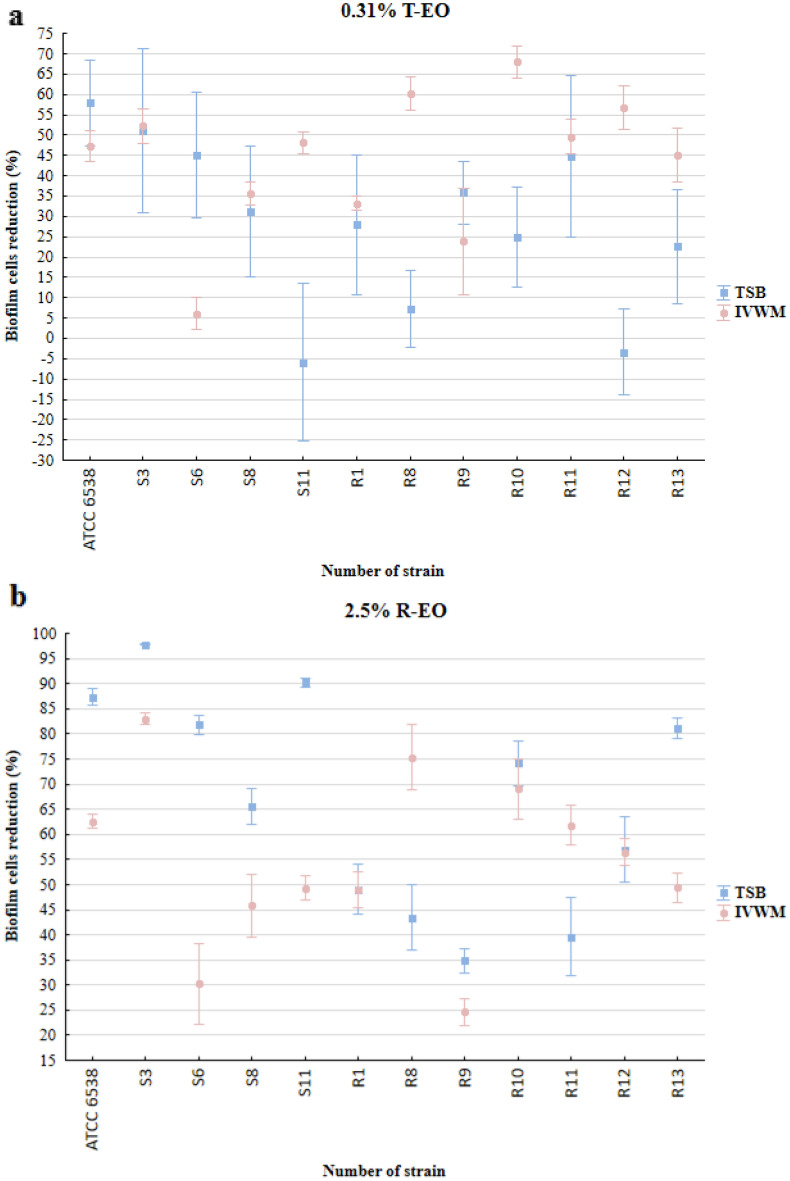
Average reduction (%) of biofilm cells of reference or clinical strains of *S. aureus* cultured in TSB medium than in IVWM after their treatment with selected concentrations (%) (v/v) of thyme Essential Oil (**a**, T-EO) and rosemary Essential Oil (**b**, R-EO). The error lines represent the standard error of the mean (n = 12).

The level of biofilm cell reduction (%) was above 90% for all strains in TSB or IVWM medium after the treatment with T-EO at concentrations of 2.5% – 0.63% (v/v) (Supplementary Figure 8A). At a T-EO concentration of 0.31% (v/v), lower reduction was observed for eight of twelve strains cultured in TSB compared to the IVWM medium (Fig. 7a). The three strains with different patterns of reduction were ATCC 6538, S6, and R9. The reduction of biofilm cells treated with R-EO was reached for all strains at concentrations ranging from 10% (v/v) to 0.63% (v/v) when cultured in TSB medium or from 10% (v/v) to 1.25% (v/v) in IVWM medium (Fig. 7b, Supplementary Figure 8B–F). At concentration of 10% (v/v) and 2.5% (v/v), a higher level of biofilm reduction was evaluated only for 17% of strains cultured in IVWM medium than in TSB. The percentage reductions of staphylococcal biofilms after treatment with selected EOs concentrations are presented in Fig. 7 and Supplementary Figure 8.

Multivariate analysis of variance was performed to evaluate the effect of medium, strain and EOs concentrations on the reduction of biofilm cells after treatment with T-EO or R-EO (Supplementary Tables 8, 9, Supplementary Figures 9, 10). In the case of both EOs, all of the factors significantly influenced the biofilm cells reduction. Additionally, for T-EO, there were significant interactions between each factor (*p* = 0.000013 for medium and strain; *p *= 0.000000 for medium and Essential Oil concentration; *p* = 0.002131 for strain and Essential Oil concentration) and all factors together (*p* = 0.000000). The biofilm cells reduction was significantly lower in TSB than in IVWM medium for T-EO (*p* = 0.000239) and significantly higher in TSB than in IVWM for R-EO (*p* = 0.000000). In the case of R-EO, significant interactions occurred between medium and strain (*p* = 0.000000), strain and the Essential Oil concentration (*p* = 0.000000), and all three factors together (*p* = 0.000000).

### *Galleria mellonella* model

Finally, the in vivo model with *Galleria mellonella* was applied to confirm the in vitro results and evaluate T-EO cytotoxicity (Supplementary Figure 11). No death larvae were observed after injection with 2.5% (v/v) T-EO emulsion or PBS. 7% of the larvae infected with *S. aureus* survived within 5 days. However, the percentage survival increased to 93% when T-EO was injected with the bacteria.

## Discussion

In this study, we applied a medium recently developed by Kadam et al. (In vitro Wound Milieu, IVWM) containing serum, cell–matrix elements, and host factors that mimic the wound environment^22^. The biofilm characteristics and eradication following EO treatment were assessed in IVWM and a standard microbiological medium (Tryptic Soy Broth, TSB).

In this research, two EOs were applied: thyme Essential Oil (T-EO) and rosemary Essential Oil (R-EO). T-EO acts against staphylococcal biofilm by targeting its two main components: biofilm-forming cells and the extracellular matrix. The hydrophilic part of thymol, the primary active ingredient in T-EO, engages with the membrane’s polar region, while its hydrophobic benzene structure and aliphatic chains penetrate the core of the biological membrane. This interaction results in significant alterations to the membrane's structure, characterized by destabilization of the lipid layer, reduced elasticity, and increased fluidity. Such changes enhance the membrane's permeability to ions like potassium and hydrogen. Moreover, the functionality of internal membrane proteins, including various enzymes and receptors, is impacted. Upon integration into the cell membrane, thymol interacts with embedded proteins through various non-specific processes, altering the conformation and function of both internal and membrane-bound proteins. Consequently, the presence of thymol leads to increased tension and destabilization of the cell membrane^23^.

Less detailed data is available regarding the mechanism behind the activity of R-EO. However, recent data published by Melkina et al. suggests that key ingredients of R-EO, such as pinene and limonene, induce the formation of reactive oxygen species, which in turn causes damage to bacterial DNA and proteins (including heat shock proteins), and also (as in case of thymol) increases membrane permeability^24^.

Our first line of research involved the use of crystal violet and TTC (2,3,5-triphenyl-tetrazolium chloride) staining to evaluate the strains' ability to form a biofilm (Fig. 1A) and the biofilm metabolic activity (Fig. 1B). The results revealed high intraspecies variability in biofilm biomass and metabolic activity, regardless of the medium used.

The biomass, metabolic activity, and cell number of *S. aureus* biofilms were higher in TSB than in IVWM medium (Fig. 1, Table 1). Unlike TSB, which primarily contains proteins, glucose, and sodium chloride, IVWM contains host antimicrobial factors (Table 4). These factors may prompt staphylococcal cells to form defensive cell aggregates, as shown in Fig. 3 and 4. This adaptation results in an altered level of tolerance towards the applied EOs, compared to biofilms cultivated in TSB, as indicated in Table 3. Studies indicate that lactoferrin inhibits bacterial growth by binding iron, which restricts its availability for bacteria, or through direct interaction with negatively charged regions of bacterial membranes, causing cell damage^25,26^. Lactoferrin can also obstruct biofilm formation by preventing its adhesion, disrupting existing structures, and significantly altering the expression of genes responsible for cell metabolism^26,27^. According to Abraham et al., non-protein compounds of bovine serum (which constitutes 70% of IVWM medium) inhibit staphylococcal biofilm formation^28^.

**Table 4:** Composition of Tryptic Soy Broth (TSB) according to the manufacturer’s specification^29^ (A). Composition of In vitro Wound Milieu (IVWM) (B).

(A) TSB^29^	
Pancreatic digest of casein	17 g/L
Peptic digest of soybean	3 g/L
Dipotassium hydrogen phosphate	2.5 g/L
Sodium chloride	5 g/L
Glucose monohydrate	2.5 g/L
(B) IVWM	
Fetal Bovine Serum	70%
Fibronectin	30–60 µg/mL
Fibrinogen	200–400 µg/mL
Lactoferrin	20–30 µg/mL
Lactic acid	11–12 mM
Collagen	10–12 µg/mL
Saline	19.6%

Moreover, the biofilm in TSB was highly confluent (Fig. 2a,c), while the biofilm cultured in IVWM formed cellular aggregates unevenly distributed on the well’s surface (Fig. 2b,d). However, these were covered with an extracellular matrix to a greater extent than the biofilms formed in the TSB (Figs. 2 and 4). These findings confirm that IVWM medium accurately reflects biofilm structure under in vivo conditions because bacterial aggregates were also observed in biopsy materials from chronic wounds^30–32^. *S. aureus* is equipped with surface proteins that bind to fibrinogen and fibronectin, leading to the formation of cell clusters. Aggregation facilitates bacterial evasion from the host’s immune system, including phagocytosis^33,34^. Nevertheless, cell aggregation has been reported to limit bacterial attachment to surfaces such as steel or hydroxyapatite. This may be explained by the fact that larger particle sizes lead to higher drag forces and lower attachment^34^. This phenomenon presumably accounts for the significantly lower biofilm mass formed in IVWM than in TSB, as demonstrated in our study.

A positive high correlation (r = 0.83) was observed between the level of biofilm biomass and metabolic activity for biofilms cultured in IVWM medium but not in TSB medium (Supplementary Table 5, Supplementary Figure 4). One explanation for the lack of correlation in TSB could be that biofilm structure is metabolically differentiated, and regions with decreased metabolic activity may be present in biofilms where an abundant extracellular matrix hinders the diffusion of nutrients and oxygen^35,36^. To extensively assess biofilm characteristics in both media, we analyzed the biofilm's 3D structure using LIVE/DEAD staining and confocal microscopy. The biofilm cells in TSB are evenly distributed, thus forming a thinner structure than in IVWM, where the cells are clustered in aggregates with more cell layers, forming bubble-like structures. However, in IVWM, there are also cell-free areas between these aggregates (Figs. 3 and 4). The different spatial distribution of cells in both media may be the cause of the lower thickness of the biofilm formed in the TSB medium than in the IVWM medium (Fig. 5). It was indicated that the percentage of live cells was higher in biofilms cultured in TSB medium than in IVWM (Table 2, Supplementary Table 6, Supplementary Figure 5E,F,G). The finding aligns with the results of the metabolic analysis assay, where the cells’ metabolic activity was higher in TSB than in IVWM medium (Fig. 1b). This may be due to less favorable conditions for biofilm formation in IVWM, such as the presence of antibiofilm compounds and a lower concentration of nutrients. When analyzing the cellular spatial composition of the biofilms, we demonstrated that in both media, the share of dead cells was higher in the top (T) than in the middle (M) and bottom (B) parts (Fig. 6, Supplementary Figure 6, Supplementary Table 7). In IVWM medium, this could be a biofilm protection mechanism against antimicrobial substances. Dead cells are peripherally localized and act as a barrier, limiting the diffusion of antimicrobials into deeper layers of the biofilm^37^.

Next, we have analyzed the properties of EOs’ emulsion as another factor that may contribute to the obtained antimicrobial effect. A recorded polydispersity index (PDI) of 0.46 for thyme Essential Oil (T-EO) and 0.68 for rosemary Essential Oil (R-EO) emulsions was high, indicating a broad size distribution of droplets within these emulsions. High PDI values typically imply a mixture of small and larger droplets, which can result from various factors such as the oils' intrinsic properties, the emulsification process, the stabilizers used, and the interaction between the oil and the aqueous phase. Multu-Inglok et al. found an inverse correlation between the size of emulsified EO droplets and their antimicrobial activity; smaller droplets correlate with higher antimicrobial potential^38^. In our study, T-EO droplets were smaller and more uniform (209 ± 22 nm) compared to R-EO droplets (995 ± 341 nm), which could partly explain why T-EO exhibited generally higher antibiofilm activity than R-EO, as shown in latter experiments.

Subsequently, *S. aureus* planktonic and biofilm cells were cultured in both media and treated with T- and R-EO. EOs showed significant anti-staphylococcal activity, though intraspecies variability was observed (Table 3, Fig. 7, Supplementary Figure 8) as well as high standard deviations from the mean. The latter was the result of the disadvantages related to the application of the 96-well plate model that we addressed, providing a sufficient number of repeats to obtain data of statistical significance^39^. Such variability and deviation from the means value are consistent with the results presented by our research team and other researchers^40–42^ and indicate the necessity of including a broad spectrum of various strains in the tests. Differences in EOs’ MIC and MBEC values were observed between experimental settings using IVWM or TSB medium, and they were higher in the case of planktonic forms than in biofilms. EOs exhibited lower antimicrobial activity against planktonic forms in IVWM than in TSB medium for more than half of the strains. This may be due to the impairment of EOs activity by bovine serum albumin^43,44^. EOs influence bacteria mainly by binding to the cell wall/membrane and leading to the disruption of its integrity. Albumin presumably binds to the hydrophobic components of EOs and hinders the interaction of EOs with bacterial membrane proteins, which decreases their efficacy^44,45^. In general, a lower level of biofilm reduction was obtained after treatment with R-EO in IVWM than in TSB medium. Regarding the antibiofilm activity of T-EO, higher cell reduction was achieved for 67% of strains cultured in IVWM than in TSB medium (Fig. 7, Supplementary Figures 8, 9 and 10).

Previous studies demonstrated that clustered bacterial cells (like those in IVWM medium) exhibit increased tolerance to antimicrobial substances^34^. Thicker biofilms, observed in IVWM medium, are also more difficult to eradicate because antimicrobial agents' penetration through the biofilm structure is reduced. The higher antibiofilm activity of T-EO in IVWM than in TSB medium against specific strains suggests that the EO influences biofilm not only by direct interaction with the cell wall but also through other mechanisms, which are enhanced with IVWM medium components.

In both the present study and our previous research, we demonstrated that key biofilm characteristics differ significantly depending on the medium in which microorganisms are cultivated^46,47^. These differences translate to variances in the results of the antimicrobial activity of the tested substances. Therefore, using only standard microbiological media in in vitro studies to evaluate the antimicrobial substances’ efficacy may lead to over- or underestimation of their effect.

Although intraspecies variability was observed, we demonstrated the high effectiveness of EOs in the partial removal of *S. aureus* biofilm and planktonic forms in both TSB and IVWM media. EOs, especially T-EO, are promising agents for the treatment of biofilm-related wound infections since, in specific cases, their activity in the IVWM medium was higher than in TSB.

We performed an in vivo model to evaluate T-EO’s cytotoxicity and antibacterial activity. The T-EO injected into larvae did not exhibit a cytotoxic effect towards them (Supplementary Figure 11). Moreover, when T-EO was injected into larvae infected with *S. aureus*, the survival rate increased dramatically from 7% to 93%. These results align with our in vitro findings and further suggest that this Essential Oil can inhibit bacterial growth, thereby preventing the progression of the infection. While this model effectively assesses the Essential Oil's in vivo activity, further animal experiments—where T-EO is applied directly to infected chronic wounds—should also be performed. These research results underscore the importance of using a medium that reflects the state of the infection site (e.g., wound exudate) and including a high number of tested strains in in vitro studies. Nevertheless, the strain number limitation is an inherent aspect of our study. Conducting the necessary analyses on a high number of strains, which often requires specialized equipment and is both time-consuming and costly, would significantly exceed the scope of this manuscript. Therefore, we adopted a pyramid-like research structure, initially performing screening tests on all strains, followed by pattern searching in a reduced number of strains. Consequently, this approach led us to focus on larvae tests with one Essential Oil (T-EO) and a single staphylococcal strain. To the best of the authors' knowledge, this study is the first to investigate the differential activity of EOs in artificial exudate (IVWM) compared to conventional medium, and it is also the first to apply a specific spectrum of biofilm-oriented methods for this purpose. We are confident that this approach will contribute to the development of effective treatments for biofilm-related infections and improve wound healing management in the future.

## Materials and methods

### Microorganisms

One reference strain, *Staphylococcus aureus* ATCC 6538 (American Type and Culture Collection), and eleven clinical isolates were tested for research purposes. The clinical strains included four MSSA strains (Methicillin-Susceptible *Staphylococcus aureus*) and seven MRSA strains (Methicillin-Resistant *Staphylococcus aureus*). The MSSA strains were marked S3, S6, S8, S11; MRSA as R1, R8-R13. The examined strains originate from the Strain and Line Collection, which belongs to the Platform for Unique Models Application, Department of the Pharmaceutical Microbiology and Parasitology, Medical University of Wroclaw. The bioethical approval was granted with the following number: Bioethical Committee of Wroclaw Medical University, protocol # 8/2016.

### Essential oils

The antimicrobial activity of two commercial Essential Oils (EOs) was tested:

o thyme Essential Oil, thyme chemotype (T-EO, obtained from *Thymus vulgaris* L. leaves), produced by Instytut Aromaterapii, Poland;
o rosemary Essential Oil, camphor chemotype (R-EO, obtained from *Rosmarinus officinalis* L. leaves), produced by Instytut Aromaterapii, Poland.

### Culture conditions

Bacteria were cultured in two media:

- Standard microbiological Tryptic Soy Broth marked TSB. The detailed composition of TSB is presented in Table 4.
- Medium prepared according to the formula presented by Kadam et al.^22^, marked IVWM (In vitro Wound Milieu).

Sterile Fetal Bovine Serum (Biowest, France, cat. No. S181H) was used as the base component of IVWM. Firstly, stock solutions of the components were prepared as follows:

- fibronectin (Human plasma fibronectin, Sigma-Aldrich, USA, cat. No. FC010) 1 mg/mL solution in autoclaved distilled water,
- fibrinogen (Fibrinogen from human plasma, Sigma-Aldrich, USA, cat. No. F3879) 10 mg/mL solution in saline (Stanlab, Poland),
- lactoferrin (Lactoferrin human, Sigma-Aldrich, USA, cat. No. L4040) 2 mg/mL solution in Dulbecco’s Phosphate Buffered Saline (Sigma-Aldrich, USA),
- lactic acid (Sigma-Aldrich, USA, cat. No. W261114) 11.4 M solution,

and they were filtered using a 0.22 ​μm syringe filter. Collagen (Collagen solution from bovine skin, concentration 2.9–3.2 mg/mL, Sigma-Aldrich, USA, cat. No. C4243) was purchased sterile. The IVWM was obtained by combining the ingredients at concentrations presented in Table 4. The medium was stored for a maximum of seven days at 2–8 °C and was protected from light.

### Assessment of EOs chemical composition using gas chromatography-mass spectrometry (GC–MS)

The experiment was performed according to the methodology presented in our previous studies^40,48^. Briefly, EOs were diluted 50 times with hexane, mixed, and analysed. Agilent 7890B GC system coupled with 7000GC/TQ, equipped with \ PAL RSI85 autosampler (Agilent Technologies, USA) and an HP-5 MS column (30 m × 0.25 mm × 0.25 μm) was used for the analysis. A total flow of helium as a carrier gas was 1 mL/min. The mode of injection was split in a ratio of 1:100. Analysis conditions were as follows: the initial temperature was 50 °C for 1 min, then increased to 4 °C/min to 170 °C, and then 10 °C/min to 280 °C which maintained for 2 min. The MS detector settings were as follows: temperature of transfer line, source, and quadrupole – 320, 230, and 150 °C, respectively, and 70 eV voltage of ionization. Detection was carried out in total scan mode at 30–400 m/z. The obtained mass spectra and retention index (RI) were compared to data from literature and the NIST 17.1 library. Indexes of linear retention were assessed with a mixture of C8–C20 saturated alkanes under the conditions applied for EOs analysis. The relative abundance of each EO component was expressed as percentage content based on peak area normalization. The MassHunter Workstation Software version B.09.00 was used for peak normalization. All analyses were performed in triplicate^40,48^.

### Evaluation of biofilm biomass, biofilm metabolic activity, and the number of colony-forming units

Biofilm mass and biofilm metabolic activity of one reference and eleven clinical *S. aureus* strains were evaluated in TSB or IVWM (In vitro Wound Milieu) medium. The number of colony-forming units was assessed for the reference strain. For this purpose, the bacteria were pre-incubated overnight in an appropriate medium (TSB or IVWM) at 37 °C. Next, the bacterial suspensions were prepared in saline and adjusted to 0.5 MF (McFarland, 1.5 × 10^8^ CFU/mL (Colony-Forming Unit)) using a densitometer and diluted thousand times in TSB or IVWM. 500 µL of this suspension was poured into the wells of a 48-well polystyrene plate and incubated for 24 h under static conditions at 37 °C. To assess the total biofilm mass, crystal violet staining was performed. The level of biofilm activity was evaluated using tetrazolium staining. Both tests were performed in two independent experiments in six replicates. Quantitative culturing was performed in one experiment in triplicate to determine the number of colony-forming units.

Evaluation of biofilm biomass level using crystal violet assay

After the biofilm culturing described above, the medium was removed, and the plates were dried at 37 °C for 10 min.

Subsequently, 500 µL of 20% (v/v) crystal violet solution in water was added to the wells, and the plates were kept at room temperature for 10 min. The stain was gently removed, the biofilm was washed once with 500 µL of saline, and the plates were incubated at 37 °C for 10 min. Next, violet crystals were dissolved with 500 µL of 30% (v/v) acetic acid water solution, and the plates were shaken for 30 min at 450 rpm at room temperature. 100 µL of the solution was transferred from one well in four replicates to 96-well plates, and the absorbance was measured at 550 nm using a spectrophotometer. The average absorbance was calculated for each sample. The absorbance of media without bacteria was also measured, and their average values were subtracted from the absorbance of each sample. Based on the results, the strains were divided into four groups according to their biofilm biomass levels:

- High biofilm biomass in TSB: S11, R12, R13;
- Low biofilm biomass in TSB: ATCC 6538, S3, R1;
- High biofilm biomass in IVWM: S6, S8, R9;
- Low biofilm biomass in IVWM: R8, R10, R11.
- Assessment of biofilm activity level using tetrazolium staining

The biofilm was cultured as presented above, and the medium was removed from above the cells. Next, metabolically active biofilm cells were two-hours stained at 37 °C with 500 µL of 0.1% (w/v) TTC solution (2,3,5-triphenyl-tetrazolium chloride, Sigma-Aldrich, USA) in TSB or IVWM medium. Next, the medium from above the biofilm was removed. The examined plates were subjected to drying (37 °C/ 10 min). 500 µL of methanol and acetic acid (9:1 ratio) solution was introduced to the wells, and the plates were shaken for 30 min at room temperature (400 rpm). 100 µL of the solution was transferred from one well in four replicates to 96-well plates, and the absorbance was measured at 490 nm using a spectrophotometer. The average absorbance was calculated for each sample. The absorbance of media without bacteria was also measured, and their average values were subtracted from the absorbance of each sample. In the macroscopic visualizations of TTC- and CV-dyed biofilms, the contrast in pictures was enhanced using GIMP software (Version 2.20.22, www.gimp.org, assessed 26.11.2020, while original images are presented in Supplementary Figure 12).

- Assessment of the number of colony-forming units

As described above, the biofilm was cultured in polystyrene plates in TSB or IVWM medium. Subsequently, the medium was removed, and each well was shaken with 500 µL of 0.1% (w/v) saponin/ water solution for 30 s at 600 rpm. Solutions from each well were resuspended and transferred to Eppendorf tubes. Plates were shaken again for 30 s/600 rpm with the fresh saponin solution (500 µL), and solutions from both steps were combined. The serial dilutions of the suspension were then prepared in saline solution and cultured onto Mueller–Hinton agar Petri dishes and incubated for 24 h at 37 °C. The CFU number was counted using ImageJ (National Institutes of Health, Bethesda, MD, USA, accessed on 1 December 2022).

### Visualization of live and dead biofilm-forming cells using fluorescent dyes and confocal microscopy

The staphylococcal strains were cultured in TSB or IVWM medium in 24-well plates. The preparation of suspensions and biofilm cultivation conditions were the same as described in the section “Evaluation of biofilm biomass, biofilm metabolic activity, and the number of colony-forming units”. Next, the biofilms were analyzed according to the methodology presented in our previous study^49^. Filmtracer™ LIVE/DEAD™ Biofilm Viability Kit (Thermo Fischer Scientific, USA) was prepared following the protocol of the manufacturer. The dye was then used to evaluate the membrane integrity and indirectly to measure a relative number of live and dead biofilm-forming cells and to visualize the morphology/spatial distribution of cells within biofilm structure. An SP8 MP laser-scanning confocal microscope (Leica, Germany) was used for the microscopic visualizations. SYTO-9 (applied to visualize live bacteria) was excited at a wavelength of 488 nm with a laser line (SP8). The collected emission was within the 500–530 nm range. Propidium iodide (PI) applied to visualize dead bacteria was excited at 552 nm wavelength with a laser line (SP8). The emission of PI was collected within the 575–625 nm SP8 ranges. 20 µm dry objectives were used in a sequence for data collection. The signal intensity and settings were set on the brightest parts to prevent oversaturation. An ImageJ (National Institutes of Health, Bethesda, MD, USA) software was used to analyze the obtained images. The captured vision field was considered the Region of Interest (ROI). In each ROI, the mean grey value (MGV) was registered for red and green fluorescence channels. It was an estimator of differences in the dead (red) and live (green) cells number. The MGV was the sum of the gray values of all the pixels in the section divided by the pixels number. The MGV was computed for RGB images by converting each pixel to grayscale with the equation: gray = 0.114 blue + 0.587 green + 0.299 red^49^.

### Visualization of biofilm using a scanning electron microscope

The *S. aureus* ATCC 6538 and R1 biofilm strains were visualized with a Scanning Electron Microscope (SEM, Auriga 60, ZEISS, Germany). Firstly, 1 mL of 2% (w/v) Bacteriological Lab Agar was poured into a 24-well plate and left for solidification. Next, 500 µL of the appropriate medium (TSB or IVWM) was poured into the wells with agar, and the plate was kept refrigerated for 24 h in order to soak the agar surface with the medium. After this time, the medium was removed from above the agar.

Subsequently, the bacterial suspensions were prepared in saline and adjusted to 0.5 MF (McFarland, 1.5 × 10^8^ CFU/mL (Colony-Forming Unit)) using a densitometer and diluted thousand times in TSB or IVWM. The suspensions were added in the amount of 500 µL to the agar wells. The plate was incubated for 24 h at 37 °C under static conditions. The medium was then removed, and 500 µL of 4.5% (v/v) glutaraldehyde was poured. The biofilms were visualised according to the methodology presented in our previous study^49^. Drying of the samples was performed with a critical point dryer EM CPD300 (Leica Microsystems, Germany). Next, with the use of EM ACE600, Leica sputter (Leica Microsystems, Germany), the samples were sputtered with Au/Pd (60:40). They were then analyzed with a Scanning Electron Microscope^49^.

### Evaluation of minimal inhibitory and minimal biofilm eradication concentrations of EOs emulsions

The antimicrobial activity of T-EO or R-EO was assessed in 96-well plates in TSB or IVWM medium. Tests were carried out in two independent experiments in three replicates. For both experiments, bacterial suspension was prepared as follows. From overnight cultures in TSB or IVWM medium suspensions were prepared in saline and adjusted to 0.5 MF (McFarland, 1.5 × 10^8^ CFU/mL (Colony-Forming Unit)) using a densitometer and diluted a thousand times in TSB or IVWM. EOs were tested as emulsions in TSB or IVWM medium with the addition of Tween 20. In the T-EO emulsions stock solution, the Essential Oil constituted 5% (v/v) of the emulsion volume, and in R-EO, 10% (v/v). Tween 20 constituted 1% (v/v) of the entire volume on both emulsions. The emulsions were prepared as follows: the EOs were mixed with Tween 20 for 30 min using a magnetic stirrer. Next, the medium was gradually added and stirred for one hour more at room temperature.

Minimal inhibitory concentration (MIC) evaluation

To evaluate MIC values, geometric concentrations of EOs’ stock solutions were prepared in TSB or IVWM medium and added in a volume of 100 µL to the wells of the plates. Next, 100 µL of bacterial suspensions in TSB or IVWM medium (prepared as described above) were added to the wells. Therefore, the actual concentration of Essential Oils tested ranged from 2.5 to 0.001% (v/v) for T-EO and from 5 to 0.0025% (v/v) for R-EO. The plates were incubated for 24 h at 37 °C with shaking at 450 rpm. Controls of bacterial growth (bacteria in TSB or IVWM medium) and medium sterility (medium only) were also prepared. After incubation, 20 µL of 1% (w/v) TTC solution (2,3,5-triphenyl-tetrazolium chloride, Sigma-Aldrich, USA) in the medium was added to each well, and plates were incubated for 2 h under the same conditions. MIC values were evaluated in the first well, where no red color was observed. The influence of five concentrations [(%) (v/v)] of Tween 20 on *S. aureus* ATCC 6538 planktonic forms cultured in TSB medium was also evaluated in the same manner as the test with EOs. In addition, the absorbance of the samples at 580 nm was measured using a spectrophotometer before adding the TTC solution. The percentage of cell viability was calculated in each Tween 20 concentration with respect to the growth control. The test was performed in one experiment in six replicates.

Minimal biofilm eradication concentration (MBEC) evaluation

To analyze the antibiofilm properties of EOs emulsions, biofilms were first cultivated in polystyrene plates. For this purpose, 100 µL of TSB or IVWM was added to the wells of the plates, and 100 µL of bacterial suspensions in TSB or IVWM medium (prepared as described above) was poured. The plates were incubated at 37 °C for 24 h under statically conditions. The geometric concentrations of the EOs’ stock solutions were then prepared in TSB or IVWM medium at concentrations ranging from 5 to 0.002% (v/v) for T-EO and from 10 to 0.005% (v/v) for R-EO. After the biofilm’s incubation, the medium was gently removed, and 200 µL of EOs emulsions was added to the wells. The plates were then incubated for 24 h (at 37 °C) under statically conditions, and the medium was gently pipetted out. Next, 0.1% (w/v) TTC solution in the medium was added to each well at the volume of 200 µL for 2 h at 37 °C. Then, MBEC values were assessed in the first colorless well.

Subsequently, the medium was removed, and 200 µL of a solution of methanol and acetic acid in a 9:1 ratio was poured into the wells, and the plates were subjected to shaking for one hour at room temperature at 550 rpm. 100 µL of the solution was transferred to fresh 96-well plates, and the absorbance of the solution was spectrophotometrically measured at 490 nm. Controls of bacterial growth (bacteria in TSB or IVWM medium) and medium sterility (medium only) were also prepared. To evaluate the percentage reduction of biofilm cells treated with EOs emulsions, the absorbance value of the sample was compared to the average bacterial growth absorbance.

### Analysis of the size of EOs emulsion droplets

The hydrodynamic diameters of the EOs droplets within emulsions were measured with the dynamic light scattering method. The analysis was performed using the Zetasizer Nano ZS ZEN3600 (Malvern Instruments Ltd., UK) with a detector position at 173° and laser light at λ = 633 nm. Emulsions of EOs were prepared in TSB medium with 1% (v/v) Tween 20 and diluted in sterile purified water one thousand times prior to the measurement. The Essential Oil phase content was 5% (v/v) in the case of T-EO and 10% (v/v) regarding R-EO. The test was run at 25 ± 0.1 °C, and the samples were measured at least 5 times. The values of hydrodynamic diameters included in the work are Z-Average values.

### *Galleria mellonella* model

The model with *Galleria mellonella* larvae was performed to assess T-EO cytotoxicity and confirm the Essential Oil’s anti-staphylococcal activity in vivo. T-EO was selected due to its higher antimicrobial activity than R-EO. This Essential Oil was tested at a concentration of 2.5% (v/v) as it was the highest assessed MBEC value. Sixth instar larvae of the greater wax moth, *G. mellonella*, with an average weight of 0.21 g, were selected for the experiment. The larvae were injected with 10 µL of 2.5% (v/v) T-EO emulsion to evaluate the Essential Oil’s cytotoxicity or with 10 µL of the *S. aureus* ATCC 6538 (American Type Culture Collection) strain at 1.25 × 10^9^ CFU/mL or with 10 µL of the *S. aureus* 6538 and 10 µL of 2.5%(v/v) T-EO (in distinct prolegs). The dose 1.25 × 10^9^ CFU/mL was used, as a gradual decrease in larval survival occurred over the 5 days with this amount. Moreover, negative control with 10 µL of PBS (Dulbecco′s Phosphate Buffered Saline, Biowest, USA) and usability control with 10 µL of 96% (v/v) ethanol were performed. The larvae were placed in 90-mm Petri dishes and incubated at 30 °C for five days. Each day, the mortality of larvae was monitored. Death was defined when the larvae were nonmobile, melanized, and did not react to physical stimuli. Three sub-groups of five larvae each (n = 15) were analyzed for each testing condition (there were 5 testing conditions: larvae treated with PBS, ethanol, T-EO, *S.aureus* alone and T-EO + *S.aureus*). It gave a total of 75 larvae applied in the experiment. Survival of larvae treated with PBS (n = 15) was considered 100% of larvae survival. On this basis, the percentage survival of larvae within other sub-groups (5 × 3 larvae each, 60 larvae a total) was calculated.

### Statistical analysis

Calculations were performed using Statistica software (Version 13; TIBCO Software Inc, Palo Alto, California, USA). The Hampel test was performed to identify outliers in the results of the biofilm biomass test, the biofilm metabolic activity assay, the biofilm thickness, and the share of live/ dead cells when all strains were analyzed together. The normality distribution and variance homogeneity were calculated with the Shapiro–Wilk and Levene tests, respectively. The *t*-test and the Mann–Whitney U test were performed to compare differences in biofilm biomass, biofilm metabolic activity, number of colony-forming units, thickness, and live/dead cell ratio between both media. The *t*-tests were used when normal distribution was determined (*p* > 0.05). A Welch’s adjusted* t*-test was used for unequal variances (*p* < 0.05). A Mann–Whitney U test was used when the normal distribution was not determined. The Pearson correlation was calculated to assess a linear correlation between biofilm biomass level and biofilm metabolic activity. Multivariate analysis of variance was performed to evaluate the effect of medium, strain, and EOs concentration on the reduction of biofilm cells after treatment with EOs. The results of statistical analyses with a significance level of *p* < 0.05 were considered significant. The graphical abstract was created with BioRender.com (BioRender Inc, Switzerland, accessed on 17 May 2023).

### Supplementary Information

Supplementary Information.

## Data Availability

All data generated or analysed during this study are included in this published article (and its Supplementary Information files). The pre-print version of this manuscript can be found in the bioRxiv https://biorxiv.org/cgi/content/short/2023.06.21.545846v1. The data presented in this study are openly available at PPM repository at https://ppm.umw.edu.pl/info/researchdata/UMW7d9f5b28c5244b14b2ca7fb652c67cc8/.
