# Supplementary material for: The chronic wound milieu changes essential oils' antibiofilm activity—an in vitro and larval model study
Source: Sci Rep. 2024 Jan 26;14:2218. doi: 10.1038/s41598-024-52424-6 (PMC10817982; doi:10.1038/s41598-024-52424-6)
Supplement: Supplementary file 1 — Supplementary Information. [file 41598_2024_52424_MOESM1_ESM.docx]

Supplementary Material

**The chronic wound milieu changes essential oils' antibiofilm activity—an in vitro and larval model study**

**Malwina Brożyna^1*^** **^†^, Bartłomiej Dudek^1^** **^†^, Weronika Kozłowska^2^, Katarzyna Malec^3^, Justyna Paleczny^1,^ Jerzy Detyna^4^, Krystyna Fabianowska-Majewska^5^, Adam Junka^1^**

^1^Platform for Unique Models Application, Department of Pharmaceutical Microbiology and Parasitology, Wroclaw Medical University, Wroclaw, Poland

^2^Division of Pharmaceutical Biotechnology, Department of Pharmaceutical Biology and Biotechnology, Wroclaw Medical University, Wroclaw, Poland

^3^Department of Drug Form Technology, Wroclaw Medical University, Wroclaw, Poland

^4^Department of Mechanics, Materials and Biomedical Engineering, Wroclaw University of Science and Technology, Wroclaw, Poland

^5^Faculty of Medicine, Lazarski University, Warsaw, Poland

*** Correspondence:**Malwina Brożyna*

[malwina.brozyna@umw.edu.pl](mailto:malwina.brozyna@umw.edu.pl)

†  These authors share first authorship.

**Supplementary Table 1**. Ingredients of T-EO **(A)** and R-EO **(B)** measured with GC-MS (Gas Chromatography-Mass Spectrometry). Components in line with Polish Pharmakopea XI standards are marked green; the ones not in line are marked red. RI - retention index, RT – retention time, T-EO- thyme essential oil, R-RO- rosemary essential oil.

| 1. **T-EO** | | | |
| --- | --- | --- | --- |
| **RI** | **RT** | **Compound** | **T-EO** |
| 925 | 8.94 | α-thujene | 0.38 |
| 932 | 9.19 | α-pinene | 0.91 |
| 948 | 9.77 | camphene | 1.06 |
| 976 | 10.75 | β-pinene | 0.31 |
| 988 | 11.19 | myrcene | 1.11 |
| 1016 | 12.19 | α-terpinene | 1.81 |
| **1024** | **12.50** | **p-cymene** | **19.20** |
| 1028 | 12.65 | limonene | 0.37 |
| 1030 | 12.70 | eucalyptol | 0.28 |
| **1058** | **13.74** | **γ-terpinene** | **9.06** |
| 1099 | 15.28 | linalool | 3.21 |
| 1147 | 17.01 | camphor | 0.62 |
| 1172 | 17.92 | borneol | 1.98 |
| 1180 | 18.23 | terpinen-4-ol | 1.02 |
| 1195 | 18.76 | α-terpineol | 0.42 |
| 1238 | 20.27 | thymol methyl ether | 0.46 |
| **1291** | **22.15** | **thymol** | **50.59** |
| 1297 | 22.36 | carvacrol | 5.65 |
| 1426 | 26.37 | caryophyllene | 1.66 |

Polish Pharmakopea XI ranges for thyme oil, thymol chemotype:

α- thujene: 0.2 percent to 1.5 percent

β-myrcene: 1.0 percent to 3.0 percent

α-terpinene: 0.9 percent to 2.6 percent

p-cymene: 14.0 percent to 28.0 percent

γ-terpinene: 4.0 percent to 12.0 percent

linalool: 1.5 percent to 6.5 percent

terpinen-4-ol: 0.1 percent to 2.5 percent

carvacrol methyl ether: 0.05 percent to 1.5 percent

thymol: 37.0 percent to 55.0 percent

carvacrol: 0.5 percent to 5.5 percent

| 1. **R-EO** | | | |
| --- | --- | --- | --- |
| **RI** | **RT** | **Compound** | **R-EO** |
| 921 | 8.81 | tricyclene | 0.41 |
| 925 | 8.94 | α-thujene | 0.11 |
| **933** | **9.22** | **α-pinene** | **21.07** |
| 948 | 9.77 | camphene | 8.81 |
| 952 | 9.90 | thuja-2,4(10)-diene | 0.30 |
| 976 | 10.76 | β-pinene | 3.78 |
| 984 | 11.04 | octen-2-ol | 0.20 |
| 989 | 11.20 | myrcene | 2.70 |
| 1005 | 11.80 | α-phellandrene | 0.52 |
| 1008 | 11.89 | 3-carene | 0.61 |
| 1016 | 12.19 | α-terpinene | 0.65 |
| 1024 | 12.48 | p-cymene | 2.46 |
| 1029 | 12.66 | limonene | 3.88 |
| **1032** | **12.79** | **1,8-cineole** | **19.98** |
| 1035 | 12.91 | trans-β-ocimene | 0.16 |
| 1057 | 13.73 | γ-terpinene | 1.01 |
| 1084 | 14.73 | terpinolene | 0.68 |
| 1099 | 15.28 | linalool | 0.71 |
| **1148** | **17.05** | **camphor** | **18.52** |
| 1172 | 17.92 | borneol | 3.54 |
| 1180 | 18.23 | terpinen-4-ol | 0.68 |
| 1194 | 18.76 | α-terpineol | 2.32 |
| 1206 | 19.18 | verbenone | 1.91 |
| 1284 | 21.88 | bornyl acetate | 1.23 |
| 1369 | 24.74 | copaene | 0.15 |
| 1419 | 26.37 | β-caryophyllene | 2.76 |
| 1456 | 27.50 | α-humulene | 0.50 |
| 1479 | 28.23 | γ-muurolene | 0.11 |
| 1517 | 29.41 | δ-cadinene | 0.12 |

For rosemary oil, Spanish type**,** the percentages are within the following ranges:

α-pinene: 18 percent to 26 percent

camphene: 8.0 percent to 12.0 percent

β-pinene: 2.0 percent to 6.0 percent

β-myrcene: 1.5 percent to 5.0 percent

limonene: 2.5 percent to 5.0 percent

cineole: 16.0 percent to 25.0 percent

p-cymene: 1.0 percent to 2.2 percent

camphor: 13.0 percent to 21.0 percent

bornyl acetate: 0.5 percent to 2.5 percent

α-terpineol: 1.0 percent to 3.5 percent

borneol: 2.0 percent to 4.5 percent

verbenone: 0.7 percent to 2.5 percent

**Supplementary Table 2.** Parameters of the tested statistic of the differences in particular staphylococcal reference (ATCC 6538, American Type Culture Collection) or clinical (S3, S6, S8, S11, R1, R8-R13) strains’ ability to form biofilm biomass between Tryptic Soy Broth (TSB) or *In Vitro* Wound Milieu (IVWM) medium. The normal distribution of the absorbance values was determined in each group (Shapiro-Wilk test, p<0.05). Depending on the variance homogeneity (Levene’s test, p<0.05), t-test (for homogeneous variances, ATCC 6538, S8, S11, R1, R8, R9, R12 strains), or Welch’s t-test (for non-homogeneous variances, S3, S6, R10, R11, R13 strains) were performed. T- values of t-test, df- degrees of freedom, p- probability level (values of p<0.05 were considered significant).

| **Biofilm biomass** | | | | | |
| --- | --- | --- | --- | --- | --- |
| **Number of strain** | \| **Average TSB** \| \| --- \| | \| **Average IVWM** \| \| --- \| | \| **t** \| \| --- \| | \| **df** \| \| --- \| | \| **p** \| \| --- \| |
| \| **ATCC 6538** \| \| --- \| | 1.1 | 0.4 | 11.6 | 22 | 0.000000 |
| **S3** | 0.9 | 0.2 | 7.9 | 16 | 0.000001 |
| **S6** | 1.4 | 0.9 | 9.0 | 15 | 0.000000 |
| **S8** | 1.3 | 0.6 | 9.6 | 22 | 0.000000 |
| **S11** | 1.6 | 0.3 | 50.0 | 20 | 0.000000 |
| **R1** | 1.0 | 0.2 | 11.2 | 21 | 0.000000 |
| **R8** | 1.3 | 0.1 | 32.0 | 20 | 0.000000 |
| **R9** | 1.2 | 0.6 | 8.1 | 22 | 0.000000 |
| **R10** | 1.2 | 0.2 | 21.3 | 15 | 0.000000 |
| **R11** | 1.2 | 0.1 | 16.2 | 12 | 0.000000 |
| **R12** | 1.5 | 0.2 | 25.7 | 21 | 0.000000 |
| **R13** | 1.6 | 0.2 | 25.3 | 13 | 0.000000 |

**Supplementary Table 3.** Parameters of the tested statistic of the differences in particular staphylococcal reference (ATCC 6538, American Type Culture Collection) or clinical (S3, S6, S8, S11, R1, R8-R13) strains’ metabolic activity of biofilms between Tryptic Soy Broth (TSB) or *In Vitro* Wound Milieu (IVWM) medium. Normal distribution of the values was determined in each group except the R1 strain (Shapiro-Wilk test, p<0.05). Depending on the variance homogeneity (Levene’s test, p<0.05), t-test (for homogeneous variances, ATCC 6538, S8, R13 strains) or Welch’s t-test (for non-homogeneous variances, S3, S6, S11, R8, R9, R10, R11, R12 strains) were performed in groups with the normal distribution of values. For the R1 strain, Mann-Whitney U test was performed. T- values of t-test, df- degrees of freedom, p- probability level (values of p<0.05 were considered significant), U and Z- values of U-test.

| **Biofilm metabolic activity** | | | | | |
| --- | --- | --- | --- | --- | --- |
| **Number of strain** | \| **Average TSB** \| \| --- \| | \| **Average IVWM** \| \| --- \| | \| **t** \| \| --- \| | \| **df** \| \| --- \| | \| **p** \| \| --- \| |
| \| **ATCC 6538** \| \| --- \| | 3.0 | 0.5 | 26.7 | 22 | 0.000000 |
| **S3** | 2.9 | 0.4 | 16.2 | 11 | 0.000000 |
| **S6** | 3.1 | 1.3 | 30.2 | 14 | 0.000000 |
| **S8** | 2.5 | 0.6 | 24.3 | 21 | 0.000000 |
| **S11** | 3.2 | 0.4 | 35.4 | 18 | 0.000000 |
| **R8** | 3.5 | 0.2 | 40.4 | 13 | 0.000000 |
| **R9** | 3.3 | 0.9 | 15.2 | 13 | 0.000000 |
| **R10** | 3.5 | 0.3 | 35.9 | 13 | 0.000000 |
| **R11** | 3.4 | 0.3 | 27.7 | 13 | 0.000000 |
| **R12** | 3.2 | 0.5 | 28.7 | 16 | 0.000000 |
| **R13** | 3.7 | 0.5 | 42.0 | 22 | 0.000000 |
|  | **Rank sum**  **TSB** | **Rank sum**  **IVWM** | **U** | **Z** | **p** |
| **R1** | 222 | 78 | 0 | 4 | 0.000037 |

**Supplementary Figure 1**. Normal probability plots for analysis of differences in particular staphylococcal reference (ATCC 6538, American Type Culture Collection) or clinical (S3, S6, S8, S11, R1, R8-R13) strains’ ability to form biofilm biomass between Tryptic Soy Broth (**A**, TSB) or *In Vitro* Wound Milieu (**B**, IVWM) medium. Red curves indicate the matching of the values to the normal distribution. P- probability level calculated with Shapiro-Wilk test. Normal distribution was considered for values of p>0.05.

**Supplementary Figure 2**. Normal probability plots for analysis of differences in particular staphylococcal reference (ATCC 6538, American Type Culture Collection) or clinical (S3, S6, S8, S11, R1, R8-R13) strains’ metabolic activity of biofilms between Tryptic Soy Broth (**A**, TSB) or *In Vitro* Wound Milieu (**B**, IVWM) medium. Red curves indicate the fitting of the values to the normal distribution. P- probability level calculated with Shapiro-Wilk test. Normal distribution was considered for values of p>0.05.

**Supplementary Figure 3**. Detailed analysis of differences in all (as a group) staphylococcal strains’ ability to form biofilm biomass or their metabolic activity between Tryptic Soy Broth (TSB) or *In Vitro* Wound Milieu (IVWM) medium. Normal probability plots for biofilm biomass in TSB (**A**) or IVWM (**B**) medium. Box and whisker plot of the absorbance values of biofilm biomass (**C**). Normal probability plots for biofilm metabolic activity in TSB (**D**) or IVWM (**E**) medium. Box and whisker plot of the absorbance values of biofilm metabolic activity (**F**). 25%-75%- interquartile range, Min-Max- a range between minimal and maximum values. Red curves indicate the fitting of the values to the normal distribution. The statistically significant differences are marked with pairs of letters a/b. P- probability level calculated with Shapiro-Wilk test. Normal distribution was considered for values of p>0.05.

**Supplementary Table 4.** Parameters of the tested statistics of the differences in all (as a group) staphylococcal strains’ ability to form biofilm biomass or their metabolic activity between Tryptic Soy Broth (TSB) or *In Vitro* Wound Milieu (IVWM) medium. Non-parametric Mann-Whitney U test was performed because the distributions were non-normal strain (Shapiro-Wilk test, p<0.05). P- probability level (values of p<0.05 were considered significant), U and Z- values of U-test.

| **Analysis of all strains as a group** | | | | | |
| --- | --- | --- | --- | --- | --- |
| **Type of compared biofilm characteristic** | **Rank sum**  **TSB** | **Rank sum**  **IVWM** | **U** | **Z** | **p** |
| **Biofilm biomass** | 28802 | 9702 | 249 | 14 | 0.000000 |
| **Biofilm metabolic activity** | 30600 | 9870 | 0 | 15 | 0.000000 |

**Supplementary Table 5.** Results of the correlation analysis of average staphylococcal strains’ ability to form biofilm biomass and average metabolic activity for strains cultured in Tryptic Soy Broth (TSB) or *In Vitro* Wound Milieu (IVWM) medium. Avr- average, SD- standard deviation, r- correlation coefficient, t- values of statistics, p- probability level (values of p<0.05 were considered significant).

|  | **Correlation of biofilm biomass and metabolic activity** | | | | | | |
| --- | --- | --- | --- | --- | --- | --- | --- |
|  | **Biofilm biomass** | | **Metabolic activity** | |  | | |
| **Medium** | **Avr** | **SD** | **Avr** | **SD** | **r** | **t** | **p** |
| **TSB** | 1.3 | 0.2 | 3.2 | 0.3 | 0.32 | 1.1 | 0.314698 |
| **IVWM** | 0.3 | 0.2 | 0.5 | 0.2 | 0.83 | 4.5 | 0.001463 |

**Supplementary Figure 4.** Scatter plots of correlations of average staphylococcal strains’ ability to form biofilm biomass and average metabolic activity for strains cultured in Tryptic Soy Broth (TSB, **A**) or *In Vitro* Wound Milieu (IVWM, **B**) medium. R- correlation coefficient, p- probability level (values of p<0.05 were considered significant). Red lines indicate 95% confidence intervals.

**Supplementary Table 6.** Parameters of the tested statistics of the differences in the number of CFU/mL (Colony-Forming Units), biofilm thickness, and percentage share of LIVE cells between staphylococcal strains cultured in Tryptic Soy Broth (TSB) or *In Vitro* Wound Milieu (IVWM) medium. Normal distribution of the values and homogeneity of variances were determined in the number of CFU/mL (Shapiro-Wilk test, Levene’s test, respectively p<0.05), therefore t-test was performed. Mann-Whitney U test was performed for biofilm thickness and share of LIVE cells because the distributions were non-normal. T- values of t-test, df- degrees of freedom, p- probability level (values of p<0.05 were considered significant), U and Z- values of U-test.

| **Analysis of other biofilm characteristics** | | | | | |
| --- | --- | --- | --- | --- | --- |
| **Type of compared characteristic** | \| **Average TSB** \| \| --- \| | \| **Average IVWM** \| \| --- \| | \| **t** \| \| --- \| | \| **df** \| \| --- \| | \| **p** \| \| --- \| |
| **Number of CFU/mL** | 9.0E+14 | 1.5E+12 | 8.4 | 4 | 0.001124 |
|  | **Rank sum**  **TSB** | **Rank sum**  **IVWM** | **U** | **Z** | **p** |
| **Biofilm thickness** | 813 | 1602 | 218 | -5 | 0.000006 |
| **Share of LIVE cells** | 1702 | 854 | 224 | 5 | 0.000003 |

**Supplementary Figure 5**. Detailed analysis of the differences in the number of CFU/mL (Colony-Forming Units), biofilm thickness, and percentage share of LIVE cells between staphylococcal biofilms cultured in Tryptic Soy Broth (TSB) or *In Vitro* Wound Milieu (IVWM) medium. Normal probability plots for the number of CFU/mL in TSB (**A**) or IVWM (**B**) medium. Normal probability plots for biofilm thickness in TSB (**C**) or IVWM (**D**) medium. Normal probability plots for a share of LIVE cells in biofilm in TSB (**E**) or IVWM (**F**) medium. Box and whisker plot of share of LIVE cells in biofilm (**G**). 25%-75%- interquartile range, Min-Max- a range between minimal and maximum values. Red curves indicate the fitting of the values to the normal distribution. The statistically significant difference is marked with a pair of letters a/b. P- probability level calculated with Shapiro-Wilk test. Normal distribution was considered for values of p>0.05.


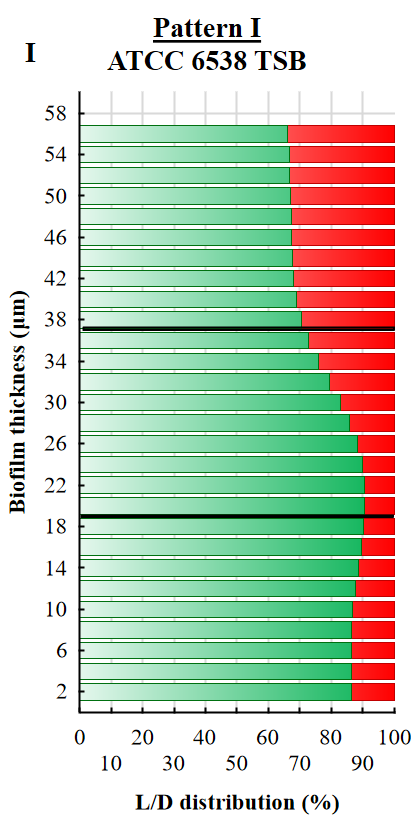

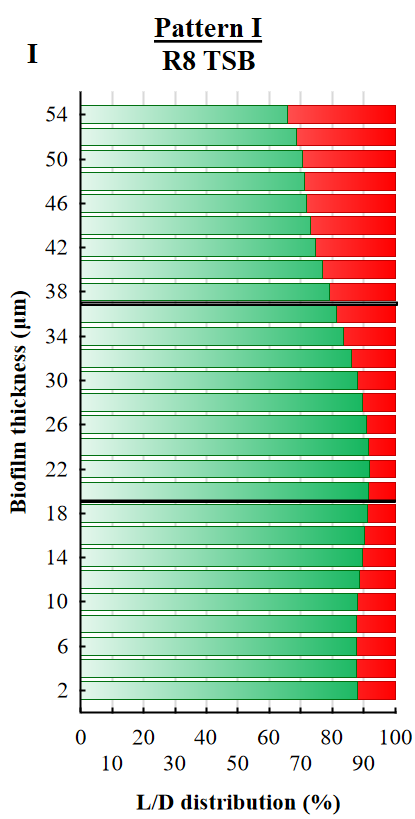

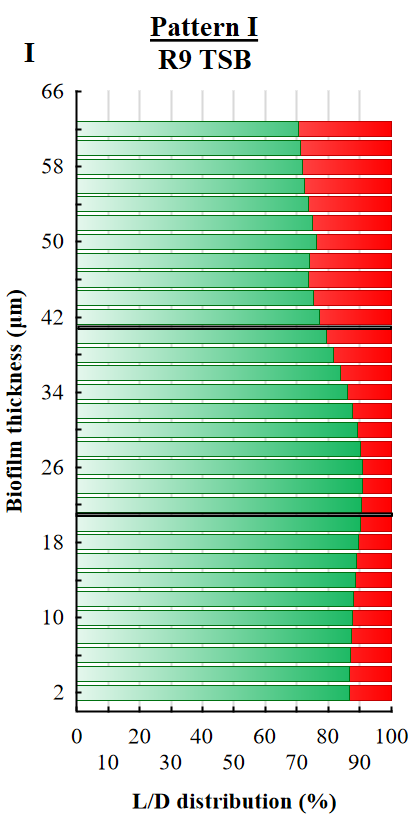

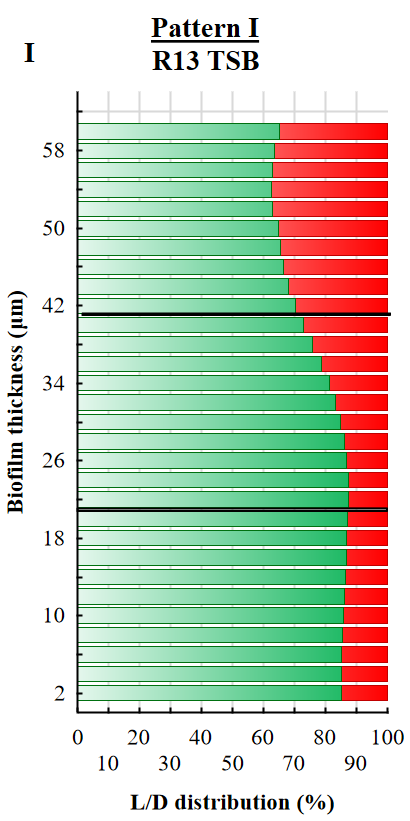

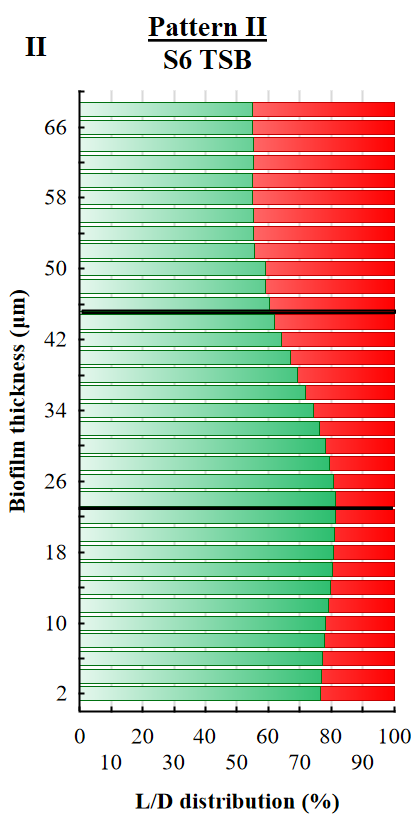

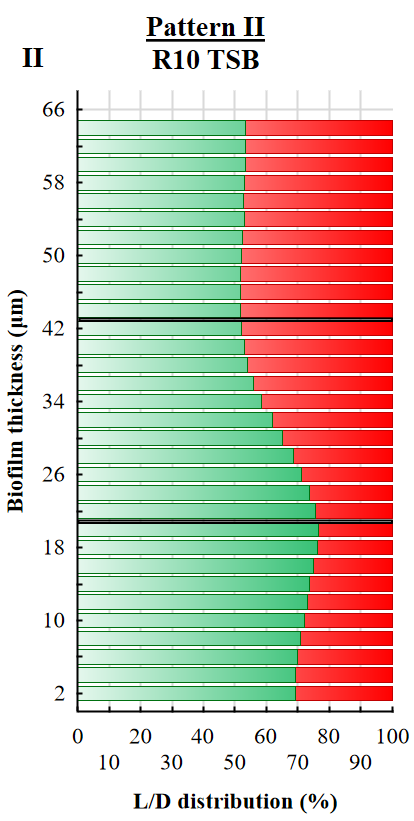


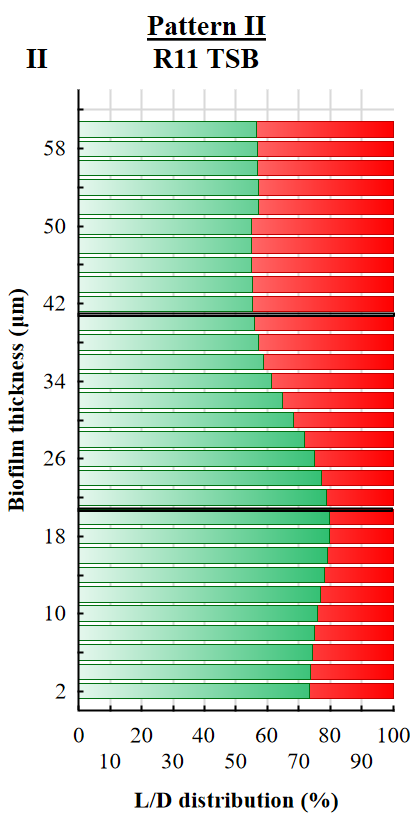

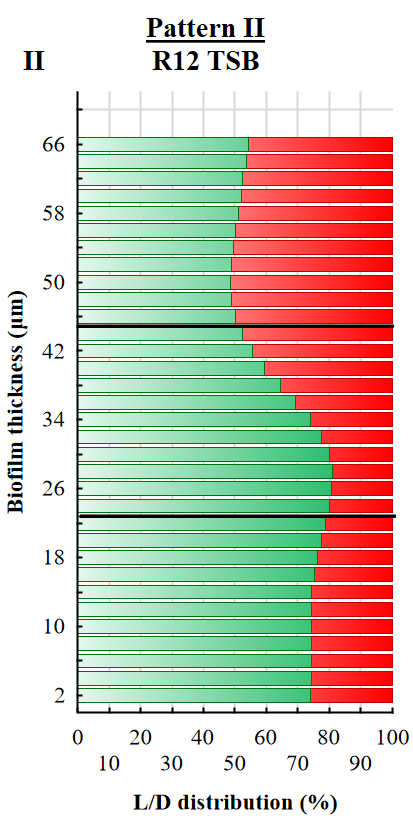


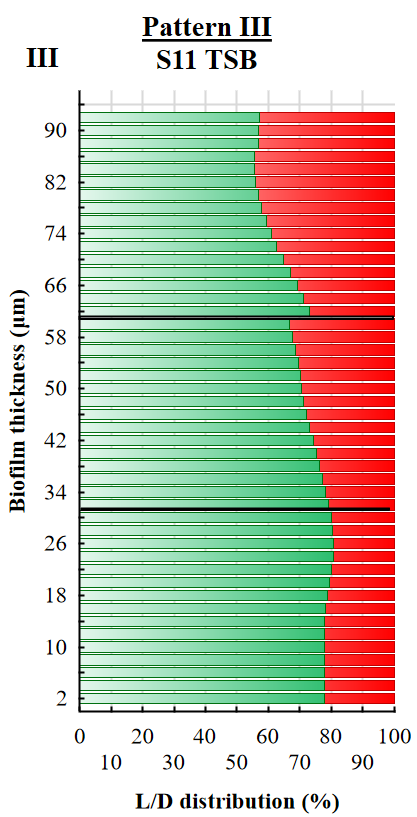

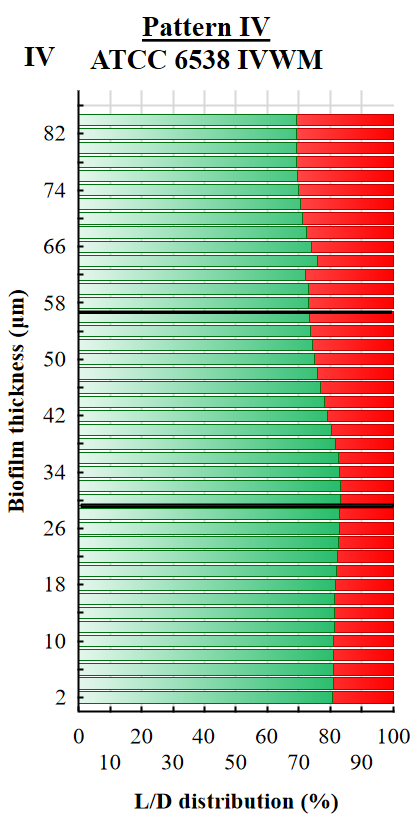

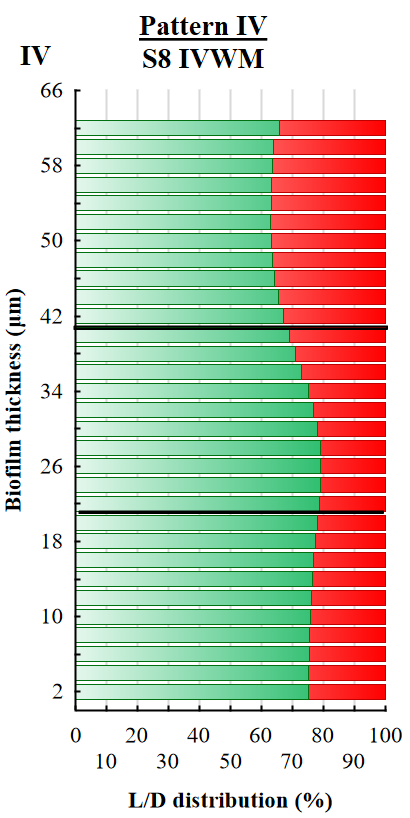

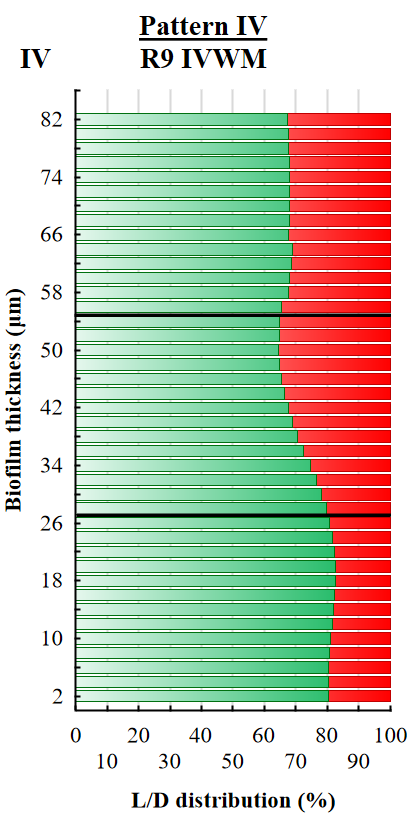


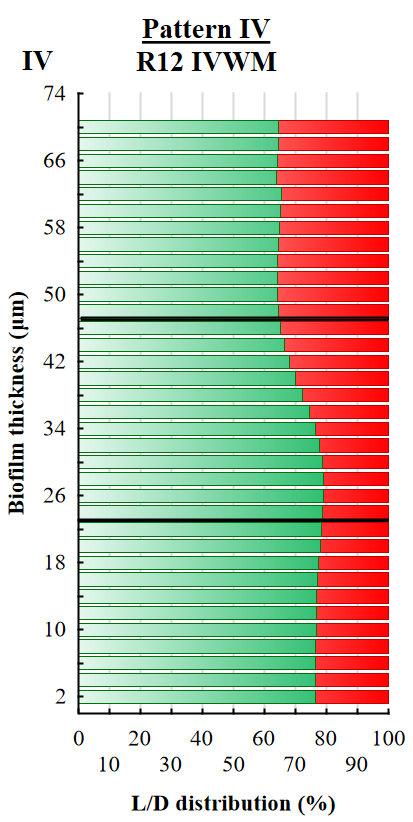

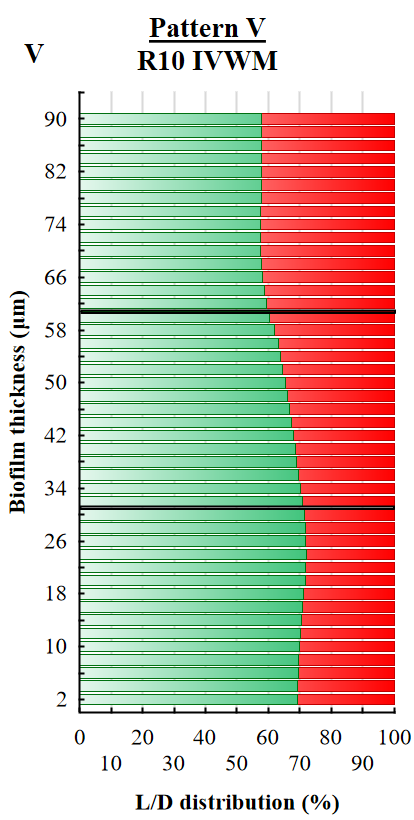

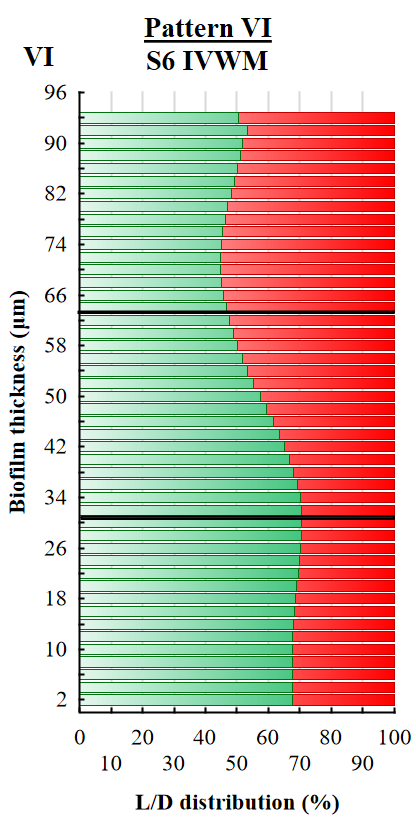

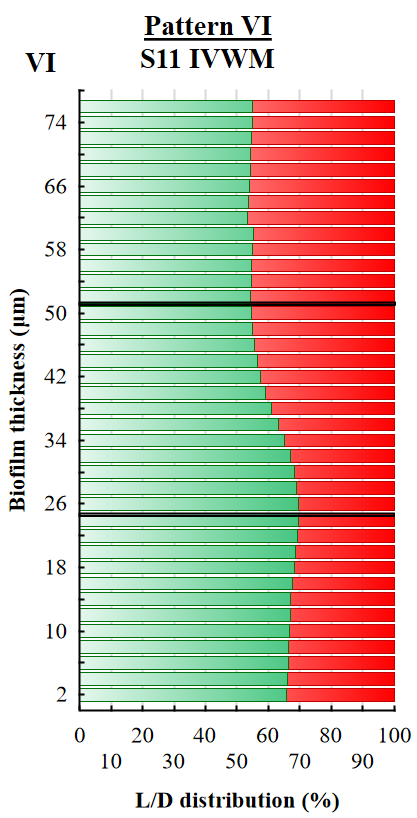


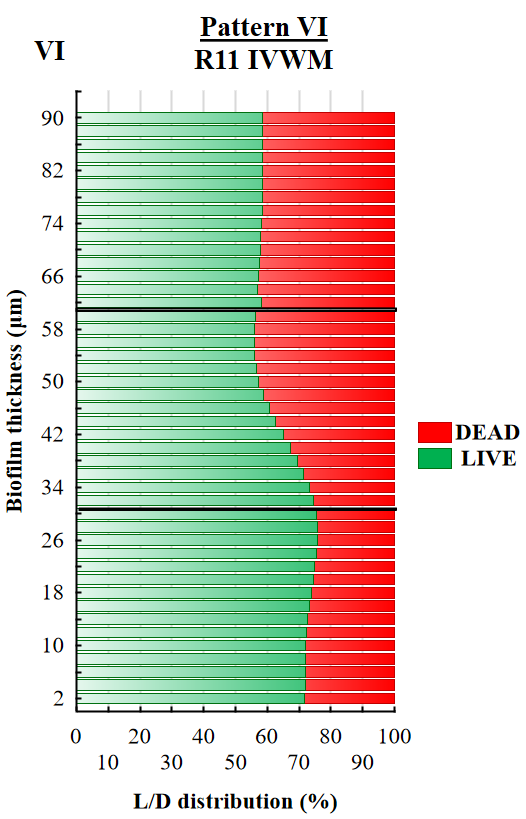


**Supplementary Figure 6.** The main patterns of live (L, green) and dead (D, red) cells distribution (%) in staphylococcal biofilms across the Z-axis, cultured in TSB (Tryptic Soy Broth) or IVWM (*In Vitro* Wound Milieu) medium. The thickness of each section was 2 µm. Black lines divide graphs into the top (T), middle (M), and bottom (B) parts. **I, II, III**- strains of particular patterns of cells share in TSB; **IV, V, VI**-strains of particular patterns of cells share in IVWM. ATCC 6538- *S. aureus* reference strain (American Type Culture Collection), S6, S8, S11, R8-R13- clinical strains of *S. aureus*. The confocal microscope SP8, magnification 25×.

**Supplementary Table 7.** Average percentage share of live and dead cells in biofilms of staphylococcal reference (ATCC 6538, American Type Culture Collection) or clinical (S3, S6, S8, S11, R1, R8-R13) strains cultured in TSB (Tryptic Soy Broth) or IVWM (*In Vitro* Wound Milieu) medium measured with a confocal microscope. Biofilms were divided into three parts along the Z-axis: top (T), middle (M), and bottom (B). The share of cells in each part was compared in both media and marked green in medium where the parameter was higher and red where it was lower.

|  | | | **Share (%) of cells in *S. aureus* biofilms** | |
| --- | --- | --- | --- | --- |
| **Strain number** | **Part of biofilm** | **Type of cells** | **TSB** | **IVWM** |
| **ATCC 6538** | T | Live | 68 | 71 |
|  |  | Dead | 32 | 29 |
|  | M | Live | 84 | 79 |
|  |  | Dead | 16 | 21 |
|  | B | Live | 87 | 82 |
|  |  | Dead | 13 | 18 |
| **S3** | T | Live | 71 | 61 |
|  |  | Dead | 29 | 39 |
|  | M | Live | 87 | 74 |
|  |  | Dead | 13 | 26 |
|  | B | Live | 85 | 76 |
|  |  | Dead | 15 | 24 |
| **S6** | T | Live | 56 | 48 |
|  |  | Dead | 44 | 52 |
|  | M | Live | 73 | 60 |
|  |  | Dead | 27 | 40 |
|  | B | Live | 79 | 69 |
|  |  | Dead | 21 | 31 |
| **S8** | T | Live | 60 | 64 |
|  |  | Dead | 40 | 36 |
|  | M | Live | 78 | 76 |
|  |  | Dead | 22 | 24 |
|  | B | Live | 84 | 76 |
|  |  | Dead | 16 | 24 |
| **S11** | T | Live | 61 | 54 |
|  |  | Dead | 39 | 46 |
|  | M | Live | 73 | 62 |
|  |  | Dead | 27 | 38 |
|  | B | Live | 79 | 67 |
|  |  | Dead | 21 | 33 |
| **R1** | T | Live | 69 | 41 |
|  |  | Dead | 31 | 59 |
|  | M | Live | 80 | 50 |
|  |  | Dead | 20 | 50 |
|  | B | Live | 85 | 60 |
|  |  | Dead | 15 | 40 |
| **R8** | T | Live | 72 | 58 |
|  |  | Dead | 28 | 42 |
|  | M | Live | 88 | 66 |
|  |  | Dead | 12 | 34 |
|  | B | Live | 89 | 71 |
|  |  | Dead | 11 | 29 |
| **R9** | T | Live | 74 | 68 |
|  |  | Dead | 26 | 32 |
|  | M | Live | 87 | 70 |
|  |  | Dead | 13 | 30 |
|  | B | Live | 88 | 81 |
|  |  | Dead | 12 | 19 |
| **R10** | T | Live | 53 | 58 |
|  |  | Dead | 47 | 42 |
|  | M | Live | 63 | 66 |
|  |  | Dead | 37 | 34 |
|  | B | Live | 73 | 71 |
|  |  | Dead | 27 | 29 |
| **R11** | T | Live | 56 | 58 |
|  |  | Dead | 44 | 42 |
|  | M | Live | 67 | 63 |
|  |  | Dead | 33 | 37 |
|  | B | Live | 77 | 74 |
|  |  | Dead | 23 | 26 |
| **R12** | T | Live | 51 | 65 |
|  |  | Dead | 49 | 35 |
|  | M | Live | 70 | 74 |
|  |  | Dead | 30 | 26 |
|  | B | Live | 75 | 77 |
|  |  | Dead | 25 | 23 |
| **R13** | T | Live | 65 | 39 |
|  |  | Dead | 35 | 61 |
|  | M | Live | 82 | 35 |
|  |  | Dead | 18 | 65 |
|  | B | Live | 86 | 51 |
|  |  | Dead | 14 | 49 |

**Supplementary Figure 7.** Average viability (%) of *S. aureus* ATCC 6538 (American Type Culture Collection) planktonic forms treated with different concentrations of Tween 20 [(%)(v/v)] in TSB (Tryptic Soy Broth) medium with regard to untreated cells. Error lines represent the the standard error of the mean . A red line marks untreated cells.

**Supplementary Figure 8**. Average reduction (%) of biofilm cells of reference (ATCC 6538, American Type Culture Collection) or clinical (S3, S6, S8, S11, R1, R8-R13) strains of *S. aureus* after treatment with selected concentrations (%) (v/v) of thyme oil (**A**, T-EO) or rosemary oil (**B-F**, R-EO). Error lines represent the standard error of the mean (n=12). TSB- Tryptic Soy Broth, IVWM- *In Vitro* Wound Milieu.

**Supplementary Table 8.** Results of the multivariate analysis of variance for biofilm cells reduction after T-EO treatment. F- values of the test, p- probability level (values of p<0.05 were considered significant).

| **Effect** | **f** | **p** |
| --- | --- | --- |
| **Medium** | 13.7 | 0.000239 |
| **Strain** | 1.9 | 0.036016 |
| **T-EO concentration (%)** | 701.9 | 0.000000 |
| **Medium and Strain** | 4.0 | 0.000013 |
| **Medium and T-EO concentration (%)** | 10.9 | 0.000000 |
| **Strain and T-EO concentration (%)** | 1.8 | 0.002131 |
| **Medium and Strain and T-EO concentration (%)** | 3.7 | 0.000000 |

**Supplementary Table 9.** Results of the multivariate analysis of variance for biofilm cells reduction after R-EO treatment. F- values of the test, p- probability level (values of p<0.05 were considered significant).

| **Effect** | **f** | **p** |
| --- | --- | --- |
| **Medium** | 83.9 | 0.000000 |
| **Strain** | 30.0 | 0.000000 |
| **R-EO concentration (%)** | 488.2 | 0.000000 |
| **Medium and Strain** | 17.1 | 0.000000 |
| **Medium and R-EO concentration (%)** | 1.1 | 0.379590 |
| **Strain and R-EO concentration (%)** | 3.1 | 0.000000 |
| **Medium and Strain and R-EO concentration (%)** | 2.6 | 0.000000 |

**Supplementary Figure 9.** Effect of medium (**A**) or medium and EO concentration (%) (v/v) interaction (**B**) on biofilm cells reduction after treatment with T-EO (thyme oil). Results of the multivariate analysis of variance. The statistically significant difference is marked with pairs of letters a/b. Tags denote means, and vertical bars denote 0.95 confidence intervals. Tags are dislocated on the X-axis values to gain higher visibility. TSB- Tryptic Soy Broth, IVWM- *In Vitro* Wound Milieu.

**Supplementary Figure 10.** Effect of medium (**A**) or medium and EO concentration (%) (v/v) interaction (**B**) on biofilm cells reduction after treatment with R-EO (rosemary oil). Results of the multivariate analysis of variance. Tags denote means, and vertical bars denote 0.95 confidence intervals. The statistically significant difference is marked with pairs of letters a/b. Tags are dislocated on the X-axis values to gain higher visibility. TSB- Tryptic Soy Broth, IVWM- *In Vitro* Wound Milieu.


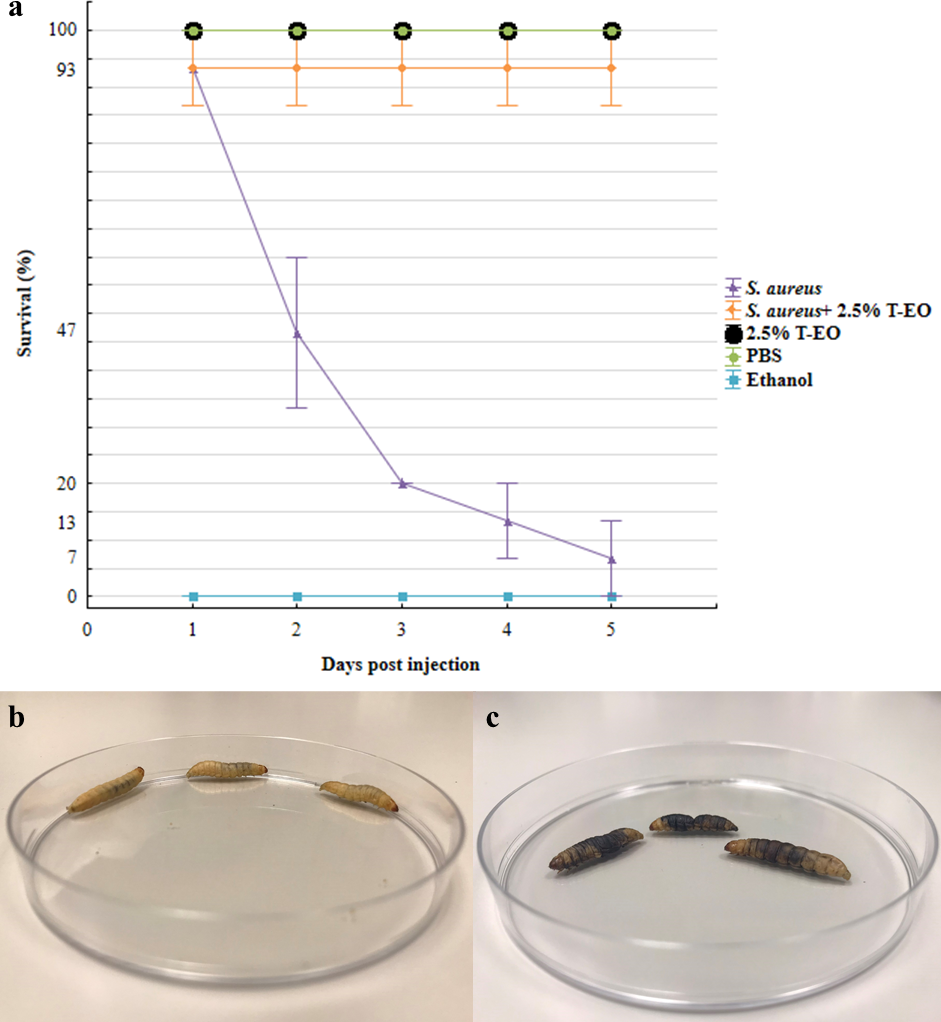


**Supplementary Figure 11. (a)**Percentage survival of *Galleria mellonella* larvae over five days. Larvae were injected with *S. aureus*ATCC 6538 strain or 2.5% (v/v) thyme oil (T-EO) or with the *S. aureus* 6538 and 2.5%(v/v) T-EO simultaneously. PBS- Phosphate Buffered Saline, negative control; Ethanol- 96% (v/v) ethanol as usability control. The error lines represent the standard error of the mean (n=75). (**b**) *Galleria mellonella* healthy, non-melanized larvae**.** (**c**) *Galleria mellonella* melanized, dead larvae.


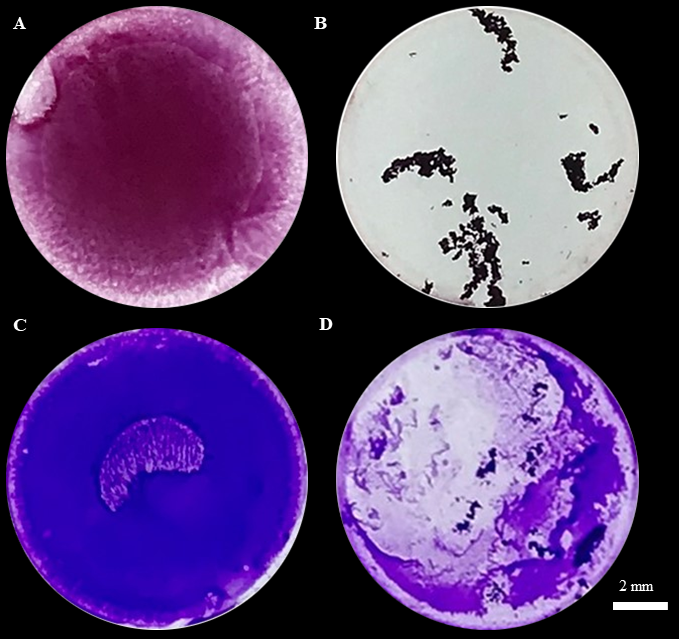


**Supplementary Figure 12**. Original images of *S. aureus* S11 biofilm formed on polystyrene. **A, B**- biofilm cultured in TSB (**A**) or IVWM (**B**) and stained with tetrazolium chloride; **C, D**- biofilm cultured in TSB (**C**) or IVWM (**D**) and stained with crystal violet. TSB- Tryptic Soy Broth, IVWM- *In Vitro* Wound Milieu. The scale bar is 2 mm.
